# Dynamic copula Bayesian network predictive model for assessing the impact of initiative programs on child undernutrition in Ethiopia, 2009–2016

**DOI:** 10.1186/s12889-025-25928-7

**Published:** 2026-01-03

**Authors:** Getnet Bogale Begashaw, Temesgen Zewotir, Haile Mekonnen Fenta, Mulu Abebe Asmamaw

**Affiliations:** 1 Department of Statistics, College of Science, Bahir Dar University, P.O. Box 79, Bahir Dar, Ethiopia; 2 Department of Data Science, College of Natural and Computational Science, Debre Berhan University, P.O. Box 445, Debre Berhan, Ethiopia; 3 School of Mathematics, Statistics and Computer Science, College of Agriculture, Engineering and Science, University of KwaZulu-Natal, Durban, South Africa; 4 Center for Environmental and Respiratory Health Research, Population Health, University of Oulu, Oulu, Finland; 5 Biocenter Oulu, University of Oulu, Oulu, Finland; 6 Atrons Technologies, Addis Ababa, Ethiopia

**Keywords:** Copula-based Models, Dependency analysis, Dynamic Bayesian network, Initiative programs, Markov Chain Monte Carlo

## Abstract

**Background:**

Child undernutrition remains a major public health concern in Ethiopia, influenced by multiple and interacting household and community factors. Despite large-scale initiatives such as the Productive Safety Net Program, Emergency Aid Program, and Health Extension Program, evidence is still needed on how these interventions affect the determinants of child nutritional status over time.

**Methods:**

We applied a Dynamic Copula Bayesian Network (DCBN) to model time-varying associations between program participation and key determinants of child undernutrition: food security (FS), household wealth (WQ), and mother subjective well-being (MSW). Data were drawn from the Young Lives–Ethiopia surveys (waves 2009, 2013, 2016) with baseline information from 2002 and 2006. The DCBN framework incorporated 26 copula families, Kendall’s τ for dependence measures, and Markov Chain Monte Carlo (MCMC) for parameter estimation. Model performance was evaluated using root mean square error (RMSE) and Nash–Sutcliffe efficiency (NSE). We further accounted for program spillovers through a community program intensity proxy and assessed robustness with baseline conditioning and inverse probability weighting (IPW).

**Results:**

Program participation was positively associated with household food security and wealth. Both FS → CUS and WQ → CUS edges showed negative and strengthening dependencies across waves, indicating that improvements in food security and wealth are associated with reductions in child undernutrition. These associations were robust to baseline conditioning, spillover adjustments, IPW weighting, and estimation method (MCMC vs. local optimization).

**Conclusions:**

The study demonstrates the utility of DCBNs for mapping dynamic, nonlinear associations between social protection and health programs and child undernutrition determinants. The results highlight that strengthening household food security and wealth plays a central role in reducing child undernutrition. Although findings are associational, the transferable dependence map can be re-estimated with contemporary data to guide program targeting, monitoring, and policy decisions in Ethiopia.

**Supplementary Information:**

The online version contains supplementary material available at 10.1186/s12889-025-25928-7.

## Introduction

Child undernutrition continues to be one of Ethiopia’s most pressing public health and development challenges, with recent national surveys indicating persistent rates of stunting, wasting, and underweight despite the implementation of major national programs such as the Productive Safety Net Program (PSNP), the Health Extension Program (HEP), and the Emergency Aid Program (EAP). More than a decade after these initiatives were scaled up, questions remain regarding how and to what extent they have influenced children’s nutritional outcomes. This study is therefore timely, as Ethiopia faces continuing shocks from climate variability, food price inflation, and service delivery gaps that exacerbate nutritional vulnerabilities.

Probabilistic modeling of temporal phenomena is of great interest in various fields due to its ability to accurately capture complex relationships and dependencies over time [1–4]. In this way, dynamic Bayesian networks (DBNs) are graphical models that model temporal dependencies and changes over time. They are probabilistic reasoning graphical models that represent temporal conditional dependencies between variables in a system [5–7]. They utilize individual nodes or component variables to capture the behavior of random variables, which enhances their usefulness in comprehending and forecasting complex phenomena, much like other probabilistic graphical models [8]. While these studies provide a foundation, details of copula functions relevant to undernutrition remain scarce. We summarize additional justification and methodological references in Supplement S1.

We apply a DCBN to map associations between initiative program participation and key determinants of child undernutrition. Common program-evaluation approaches—matching/weighting, difference-in-differences, instrumental variables, and synthetic control—are designed for causal effects under specific assumptions (ignorability, parallel trends, valid instruments, no interference) and typically model one exposure–one outcome at a time. While useful, these approaches often struggle to capture nonlinear, non-Gaussian, and time-varying dependencies across multiple interrelated factors. In contrast, DCBN provides a probabilistic, time-varying map of dependencies among multiple nodes, accommodates ordinal and skewed marginals, and models nonlinear associations. Rank-based Kendall’s τ supports robust screening and interpretable estimation. This makes it especially well-suited to complex nutritional systems where household wealth, food security, and mother wellbeing interact dynamically.

However, these existed Dynamic Bayesian network methods have limitations due to their assumption of conditional independence between variables, given their parents in the network, which may not be applicable in real-world scenarios where variables are correlated [4, 9]. This limitation can be addressed by extending them to dynamic copula Bayesian networks (DCBNs), which incorporate copulas to model complex dependencies more accurately [8]. Copulas capture the joint distribution of variables separately from their marginal distributions, providing a more accurate model of dependencies [10, 11]. This approach is widely used to model the dependence structure of two or more random variables.

Copulas are a statistical technique that separates the choice of univariate marginals from the dependence function, enabling the modeling of complex, real-valued distributions [12]. This separation provides a more accurate representation of the dependence structure between variables, making copulas useful for multivariate longitudinal data [13, 14]. Copula models excel at capturing the dependence structure between multiple variables, even when nonlinear, making them advantageous for dealing with diverse data types such as child undernutrition.

The copula Bayesian network is widely utilized in the fields of environmental science [15–19], finance [20–22], and public health [23–26], yet its application in the context of the child undernutrition literature has been insufficiently explored. Although copulas have been partially utilized in child undernutrition studies without integration with Bayesian networks, there is potential for broader adoption. A study on anemia and malnutrition among children under five years of age in Angola, Senegal, and Malawi has applied copula geo-additive models, indicating the potential for broader use of copulas in this area [27]. Likewise, a study from India examined the effect of crop diversification on undernutrition using a bivariate copula function based on simulation and revealed a significant influence of diversification on reducing undernutrition [28]. Another study proposed an optimization-based method for estimating copula parameters, which could be adapted for undernutrition research to better understand the dependencies between different variables [29]. While these studies provide a foundation, the specific types of copula functions that have proven significant in undernutrition research are not detailed in the available literature. This paper introduces a novel approach that integrates dynamic copulas into a Bayesian network framework to model the complex dependencies between initiative programs and covariates that affect children’s undernutrition status.

This research extends the DBN to the DCBN framework by incorporating copulas, enhancing the accuracy of capturing dependencies between variables. This approach allows for better representation of complex relationships, even when conditional independence assumptions are not met, offering a more robust and flexible approach to network structure [8, 30]. DCBN is utilized in various fields, such as financial risk management and climatological, hydrological, environmental, and healthcare fields, to analyze and predict the behavior of interconnected variables over time [12, 23, 31, 32]. Over a dozen copula families have been utilized in these fields, but there are limited resources for their application with Bayesian networks of child undernutrition. By applying DCBN to this domain, our study makes a methodological contribution by demonstrating how dynamic copulas can be operationalized to uncover hidden dependencies in child nutrition research, a setting where traditional statistical approaches have been limited.

Therefore, our model is applied to longitudinal Young Lives (YL) data collected in Ethiopia. The dataset includes three intervention programs, the Productive Safe Net Program (PSNP), the Health Extension Program (HEP), and the Emergency Aid Program (EAP), aimed at providing cash transfers, food assistance, and primary healthcare services to vulnerable households [33–35]. This study quantifies the dependence structure of factors on child undernutrition using the Bayesian framework to estimate model parameters. The Bayesian framework incorporates prior information about model parameters, potentially leading to more robust inferences. Furthermore, understanding the complex interrelationships between various factors influencing child undernutrition is crucial for designing effective interventions and improving child health outcomes.

We used the 2009–2016 Young Lives waves to map dynamic associations among food security, wealth, mother well-being, and child undernutrition. This study aims to map time-varying associations between initiative program participation and key determinants of child nutritional status in Ethiopia over 2009, 2013, and 2016. The specific objectives of this study are to (i) quantify time-varying dependencies among household wealth, food security, mother wellbeing, and child nutritional status in Ethiopia (2009, 2013, 2016) using a dynamic copula Bayesian network; (ii) assess how participation in national initiatives (PSNP, HEP, EAP) is associated with these pathways; and (iii) incorporate baseline data (2002/2006) and a community program intensity proxy to strengthen contextualization.

Importantly, the findings of this study are not only theoretical but also practical. By generating dependency maps between program participation and household outcomes, the results provide policymakers with a transferable framework that can be re-estimated with current survey or administrative data. This enables targeted monitoring and adjustment of Ethiopia’s nutrition and social protection programs, helping decision-makers identify which pathways—such as strengthening food security or improving household wealth—are most strongly associated with reductions in child undernutrition.

## Methods

### Conceptualizations

The PSNP in Ethiopia, launched by the Ministry of Agriculture under the umbrella of the National Food Security Program, aims to reduce chronic food insecurity and vulnerability in rural areas [36] and address short-term shocks, particularly droughts [37]. The program provides direct support to vulnerable households through cash and food transfers, public works programs, and afforestation, using a community-based targeting system [38, 39]. It aims to replace emergency assistance with a resilience-building approach for chronically food insecure households and areas receiving humanitarian aid [40].

On the other hand, the Emergency Aid Program (EAP) in Ethiopia is a humanitarian intervention system that provides life-saving support to affected communities in response to both acute and chronic crises, including natural disasters and conflicts and population displacement. Ethiopia faces challenges such as droughts, food insecurity, and poverty, leading to high child undernutrition rates. It provides food aid, enhances healthcare access, and promotes community empowerment. It educates mothers on nutrition and childcare practices and supports local initiatives for improved food security and livelihoods.

Similarly, the HEP is a community-based healthcare system launched to address health issues, especially in agrarian areas. It employs health extension workers (HEWs), typically women, to provide primary healthcare services, including maternal and child health, family planning, hygiene, immunization, and disease prevention [41, 42]. The program also addresses child undernutrition by providing nutrition education, promoting breastfeeding, monitoring growth, and managing malnutrition cases.

### Survey data: sourcing and preprocessing

For this study, we utilized YL data collected from Ethiopian children aged 1 to 15 years. The dataset spans five distinct time points: 2002, 2006, 2009, 2013, and 2016. The study involved interviewing randomly selected households to determine their participation in the PSNP, EAP, and/or HEP programs in the past year, aiming to identify program beneficiaries. The categorization of beneficiaries in the PSNP and EAP began in 2009 (third wave), while HEP categorization started in 2013 (fourth wave). As a result, data from the first and second waves (2002 & 2006) were excluded from the analysis due to the absence of program initiation during these periods. Although program participation could not be defined in 2002 and 2006, we leveraged those waves as pre-intervention baselines. Specifically, we extracted child nutritional status (CUS), household food security (FS), wealth quantile (WQ), and available household/mother covariates from 2002/2006 and used them as exogenous baseline predictors for the initial (2009) time slice. This preserves the dynamic program structure from 2009 onward while conditioning the initial slice on pre-intervention information. FS was derived using the standardized Household Food Insecurity Access Scale (HFIAS) methodology [43]. The indicator is based on nine occurrence and frequency-of-occurrence questions that capture anxiety about food supply, insufficient food quality, and insufficient food intake over the preceding four weeks. For easier analysis, these intervention programs were combined into a single variable called “programme participation status”, resulting in eight distinct categories (C, P, E, H, PE, PH, EH, and PEH), as detailed in Table 1 [9]. Given that the goal of this paper is to model the dependency of these intervention programs on factors associated with child undernutrition, we employed a dynamic copula Bayesian network model.

**Table 1:** Classification and percentage of household programme beneficiaries over time in Ethiopia

Intervention	Description	PSNP	EAP	HEP	2009	2013	2016
C	Control	No	No	No	65.0%	14.0%	16.0%
P	PSNP only	Yes	No	No	22.0%	0.4%	0.2%
E	EAP only	No	Yes	No	9.0%	1.0%	0.0%
H	HEP only	No	No	Yes	0.0%	54.0%	51.0%
PE	PSNP & EAP only	Yes	Yes	No	4.0%	0.0%	0.1%
PH	PSNP & HEP only	Yes	No	Yes	0.0%	14.0%	9.0%
EH	EAP & HEP only	No	Yes	Yes	0.0%	12.0%	20.0%
PEH	PSNP, EAP & HEP	Yes	Yes	Yes	0.0%	4.0%	4.0%
PSNP	P + PE + PH + PEH				26.0%	19.0%	13.0%
HEP	H + EH + PH + PEH				0.0%	85.0%	83.0%
EAP	E + EH + PE + PEH				13.0%	18.0%	24.0%

In 2009, H, PH, EH, and PEH were zero since HEP was not launched at that point in time

This research examined the nutritional status of children by utilizing WHO standards and standardized Z scores. Similarly, as outlined in [44], the anthropometric data of the children were documented in eight states: normal (N), underweight (U), stunted (S), wasted (W), both underweight and stunted (US), both underweight and wasted (UW), both stunted and wasted (SW), and all underweight, stunted, and wasted (USW).

### Baseline, selection-bias, and spillovers mitigation

To address pre-2009 comparability and potential selection, we implemented: (i) Baseline conditioning—including 2002/2006 CUS, FS, WQ, household size, and mother covariates as exogenous parents of the 2009 nodes; (ii) Pre-trend/balance diagnostics—standardized mean differences and rank-based tests comparing households that later participated (by 2009) versus non-participants; and (iii) Inverse-probability weighting (IPW)—stabilized weights estimated using only pre-2009 covariates and applied when estimating dependence (Kendall’s τ and copula parameters). To represent indirect program benefits, we added a leave-one-out community program intensity (${\mathrm{CPI}}_{t}$) as an exogenous node with candidate links to ${\mathrm{PS}}_{t},{\mathrm{FS}}_{t},{\mathrm{WQ}}_{t},{\mathrm{MSW}}_{t}$, and ${\mathrm{CUS}}_{t}$; uncertainty was quantified with community-block bootstrap. Results are interpreted as time-varying associations. Full derivations, weighting formulas, and the CPI construction appear in Supplement S2. In general, pre-2009 selection was probed via (i) baseline conditioning, (ii) pre-trend/balance diagnostics, and (iii) IPW sensitivity; detailed results are in Supplement S2.

To represent indirect program benefits, we constructed a community program intensity variable (${\mathrm{CPI}}_{t}$) defined as the leave-one-out proportion of other households in the respondent’s community receiving any initiative at time t (program-specific versions are detailed in Supplement S3). ${\mathrm{CPI}}_{t}$ was entered into the DCBN as an exogenous node with candidate edges to ${\mathrm{PS}}_{t},{\mathrm{FS}}_{t},{\mathrm{WQ}}_{t},{\mathrm{MSW}}_{t}$, and ${\mathrm{CUS}}_{t}$ identified through score-based structure learning. Uncertainty was quantified using community-block bootstrap. Full derivations, weighting formulas, and CPI construction details are provided in Supplements S3.

### Flow of probabilistic modeling in dynamic copula Bayesian networks

**Fig. 1:**
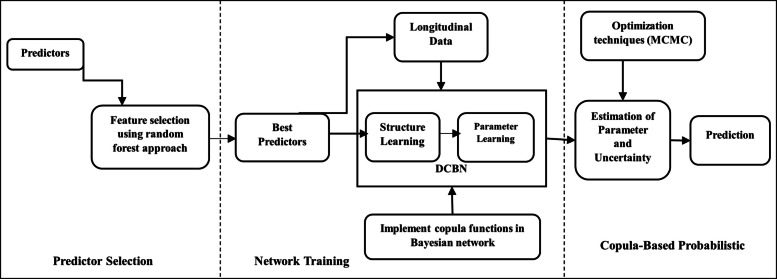
Technical flowchart of dynamic copula Bayesian network

**Fig. 2:**
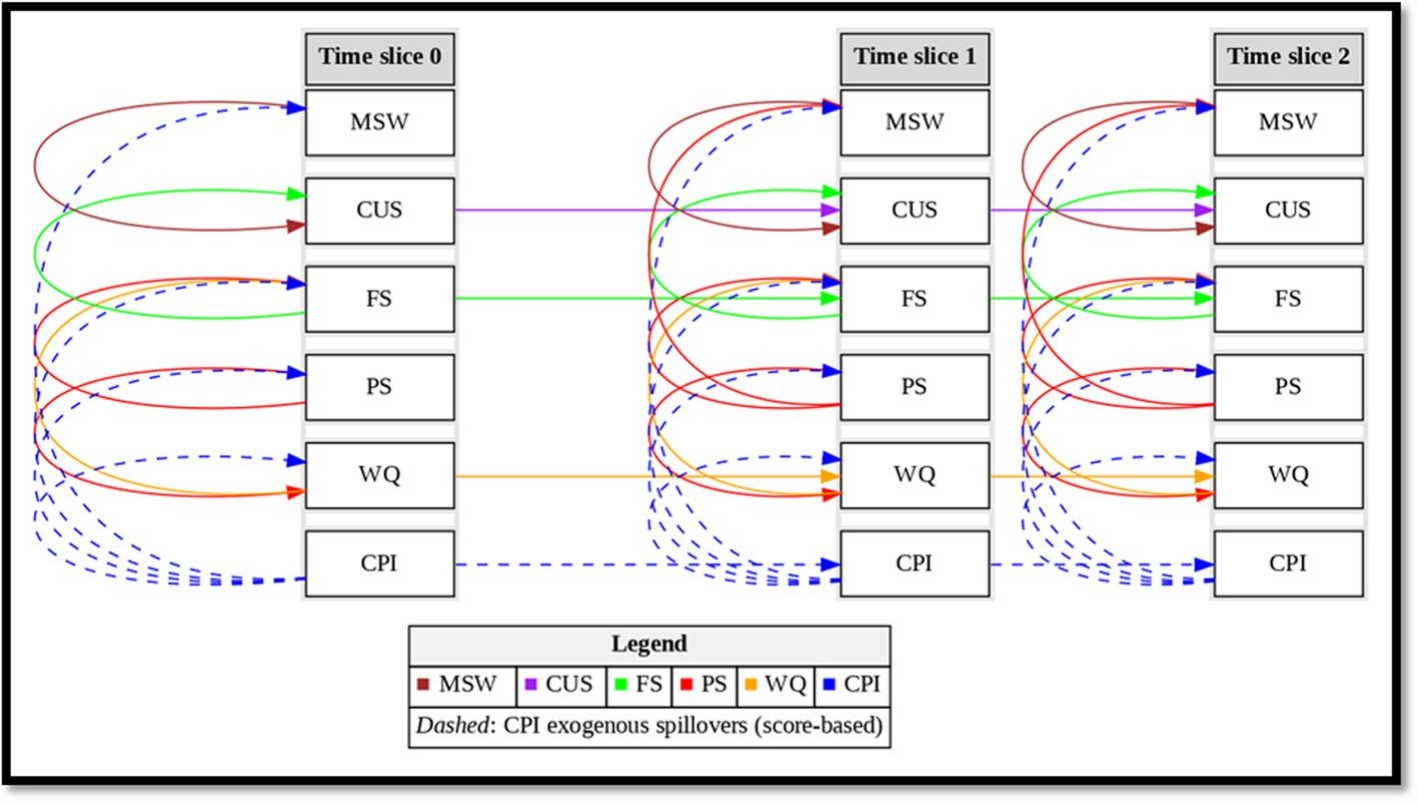
Representation of the dynamic structured probabilistic graphical model (DCBN). NB: MSW stands for mother’s subjective wellbeing, CUS is child undernutrition status, FS is food security status, PS is program participation status of the household, and WQ is wealth quantile. ${\mathbf{C}\mathbf{P}\mathbf{I}}_{{\boldsymbol{t}}}$ denotes leave-one-out Community Program Intensity at time t (spillover proxy); dashed arrows indicate exogenous spillover links selected by score-based learning. In the depicted diagram, the time slices labeled 0, 1, and 2 correspond to the years 2009, 2013, and 2016, respectively, within the dataset. Slice 0 (2009) is conditioned on 2002/2006 baselines (CUS, FS, WQ, household & mother covariates). Variable orientation: higher FS/WQ = better; higher CUS = more undernutrition. At time slice 0, the representation is denoted as ${\mathcal{G}}_{0}$. For instance, the WQ can be expressed as ${\boldsymbol{P}}\left({{\boldsymbol{x}}}^{(0)}={{\boldsymbol{W}}{\boldsymbol{Q}}}^{0}|{{\boldsymbol{P}}{\boldsymbol{S}}}^{0}\right)$, while at time slices 1 and 2, the representation is denoted as ${{\boldsymbol{G}}}_{\to }$. For example, the WQ at time slice 2 is represented as ${\boldsymbol{P}}\left({{\boldsymbol{x}}}^{(1:2)}={{\boldsymbol{W}}{\boldsymbol{Q}}}^{2}|{{\boldsymbol{P}}{\boldsymbol{S}}}^{2},{{\boldsymbol{W}}{\boldsymbol{Q}}}^{1}\right)$

As shown in Fig. 1, we initially conducted feature selection using the random forest approach, considering features with a mean decrease in accuracy exceeding the median value [45–47]. Subsequently, these selected features were fed into the Bayesian network analysis (refer to Table 4 and Figure S1 in the Supplement). In this analysis, we employed a structural learning algorithm to infer the potential edges of the program participation status (PS), which affects the nutritional status of all interns. This process yielded a directed acyclic graph (DAG), as shown in Fig. 2. After establishing the network structure, we utilized copula functions within the Bayesian framework for parameter learning. By employing both local and MCMC optimization techniques, we assessed their efficiency in estimating parameters and uncertainties and their convergence speed.

### Nodes in the copula Bayesian network

In the following DAG representing the DBN or DCBN (Fig. 2), there are 15 nodes, with each node symbolizing a distinct aspect of the modeled system. The network comprises a total of 23 directed arcs or edges, depicting the interconnections and dependencies among these nodes. The DBN encompasses a vast configuration space, with a total of 32,768 possible joint configurations of all variables. This calculation arises from each variable having two possible states, resulting in a combinatorial space of 2^15^.

We use Kendall’s τ as our dependence summary because it (i) is nonparametric and rank-based, requiring no Gaussian or linear assumptions; (ii) is invariant to monotone transformations and robust to outliers and ties, which suits our ordinal/binary household and nutrition measures; and (iii) aligns naturally with copula modeling, where τ has closed-form relationships to copula parameters for many families (e.g., Archimedean), aiding family screening, diagnostics, and stable initialization for local/MCMC optimization. By contrast, Pearson’s r targets linear, moment-based association under approximate normality, and Spearman’s ρ—while rank-based—tends to have larger small-sample variance and fewer direct parameter links for copulas. Kendall’s rank is particularly useful for detecting monotonic dependencies, making it suitable for noncontinuous data and scenarios with tied values [48]. We therefore report τ for temporal edges (Table 3) and use it to cross-check the direction and strength of estimated copula dependencies. Importantly, to ensure consistent interpretation, we harmonized signs so that higher FS = better food security, higher WQ = greater wealth, and higher CUS = more undernutrition. All reported Kendall’s τ and copula parameters reflect these directions.

### Statistical methods

In DCBN, the joint density of the entire temporal sequence can be represented using copulas. Let us denote the set of random variables as $X$ and the replication of this set for different times 0 to $T$ as ${X}^{(0:T)}=\left\{{X}^{(0)},{X}^{(1)},\dots ,{X}^{(T)}\right\}$.

The joint density of the entire temporal sequence ${x}^{(0:T)}$, i.e., ${X}^{(0)}=\left[{X}_{1}^{(0)},{X}_{2}^{(0)},\dots ,{X}_{n}^{(0)}\right]\text{ to }{X}^{(T)}=\left[{X}_{1}^{(T)},{X}_{2}^{(T)},\dots ,{X}_{n}^{(T)}\right]$ can be represented using copula-based building blocks as follows [8]:

$$P\left({x}^{(0:T)}\right)=P\left({X}^{(0)},{X}^{(1)},\dots ,{X}^{(T)}\right)=P\left({x}^{(0)}\right)\prod_{t=1}^{T} P\left({X}^{(t)}\mid {X}^{(t-1)}\right)$$

The joint density of the entire temporal sequence ${x}^{(0:T)}$ is the product of the initial density $P\left({x}^{(0)}\right)$ and the transition density $P\left({x}^{(t)}\mid {x}^{(t-1)}\right)$ for each time step $t.$ The initial density $P\left({x}^{(0)}\right)$ represents the probability density function of the variables at time $t=0$ without conditioning on any previous time step. The transition densities $P\left({x}^{(t)}\mid {x}^{(t-1)}\right)$ represent the conditional probability density functions of the variables at each time step $t$ given the variables at the previous time step $t-1$. This conditional density can be constructed using copulas, which capture the dependence structure between the variables.

Similarly, for the initial density $P\left({x}^{(0)}\right)$, we can use a copula-based approach. However, since there is no previous time step to condition on, we typically specify the joint density directly using the copula function and the marginal distributions of the variables. The joint density $P\left({x}^{(0)}\right)$ can be expressed as:

$$P\left({x}^{(0)}\right)=\prod_{i\in x}{R}_{{c}_{i}}^{(0)}\left({F}_{{x}_{i}},\left\{F{\left({x}_{{\mathrm{Pa}}_{i}}\right)}^{{\mathcal{G}}_{0}}\right\}\right)\cdot {f}_{i}\left({x}_{i}\right)$$

where ${R}_{{c}_{i}}^{(0)}$ is the copula ratio for variable ${X}_{i}$ at the initial time step, ${\mathcal{G}}_{0}$ is a DAG representing independencies over ${X}^{0}$, ${F}_{{x}_{i}}$ is the cumulative distribution function of variable ${X}_{i}$, $F{\left({x}_{{\mathrm{Pa}}_{i}}\right)}^{{\mathcal{G}}_{0}}$ represents the cumulative distribution function of the parents of variable ${X}_{i}$ as defined in the DAG ${\mathcal{G}}_{0}$, and ${f}_{i}\left({x}_{i}\right)$ is the probability density function of variable ${X}_{i}$.

The transition density $P\left({X}^{(t)}\mid {X}^{(t-1)}\right)$ represents the conditional probability density function of the variables at time $t$ given the variables at time $t-1$. In DCBN, this transition density can also be expressed using copula functions and conditional densities. Using copulas, the transition density can be written as:

$$P\left({x}^{(t)}\mid {x}^{(t-1)}\right)=\prod_{i=1}^{n} {c}_{i}\left({F}_{{x}_{i}^{(t)}}\mid \left\{F{\left({x}_{{\mathrm{Pa}}_{i}}^{(t)}\right)}^{{G}_{\to }}\right\}\right)\cdot {f}_{i}\left({x}_{i}^{(t)}\right)$$

where ${G}_{\to }$ is a DAG defined over a two-replication set of random variables ${X}_{i}$ and ${X}_{j}$, ${c}_{i}\left({F}_{{x}_{i}^{(t)}}\mid \left\{F{\left({x}_{{\mathrm{Pa}}_{i}}^{(t)}\right)}^{{G}_{\to }}\right\}\right)$ represents the local copula density for variable ${X}_{i}$ at time $t$ conditioned on the values of its parents at time $t$ as defined in the DAG ${G}_{\to }$, ${F}_{{x}_{i}^{(t)}}$ is the cumulative distribution function of variable ${X}_{i}$ at time $t$, $F{\left({x}_{{\mathrm{Pa}}_{i}}^{(t)}\right)}^{{G}_{\to }}$ represents the cumulative distribution function of the parents of variable ${X}_{i}$ at time $t$ as defined in the DAG ${G}_{\to }$, and ${f}_{i}\left({x}_{i}^{(t)}\right)$ is the probability density function of variable ${X}_{i}$ at time $t$.

We do not assume Gaussian outcomes. For each node and time slice, we estimate appropriate marginals—Bernoulli (binary), ordered-logit or empirical CDF (ordinal), and flexible continuous distributions (continuous) —and map observations to $[\mathrm{0,1}]$ via the probability integral transform. For discrete/ordinal variables we use the distributional transform to obtain pseudo-uniforms with correct tie handling. This separation of marginals from dependence allows heavy tails, skewness, and mixed scales without linear/Gaussian assumptions. Dependence is modeled with slice-specific copulas (${\theta }_{t}$ for $t=\mathrm{2009,2013,2016}$), permitting non-stationary associations over time. The DCBN includes autoregressive edges (e.g., ${\mathrm{CUS}}_{t-1}\to {\mathrm{CUS}}_{t},{\mathrm{FS}}_{t-1}\to {\mathrm{FS}}_{t},{\mathrm{WQ}}_{t-1}\to {\mathrm{WQ}}_{t}$) to capture persistence in each process. Feedback is allowed across time but the graph is acyclic within each slice.

### Structure learning

This study uses the standard greedy search approach to learn the structure of ${\mathcal{G}}_{0}$ and ${G}_{\to }$ in a DCBN by applying local structure modifications such as adding, deleting, or reversing edges [8] based on the model selection score, which in this case is the Bayesian information criterion of Schwarz [49]. The BIC balances the likelihood of the data given the model with the complexity of the model [8]:

$$\mathrm{score}(\mathcal{G}:\mathcal{D})={\ell}(\mathcal{D}:\widehat{\theta },\mathcal{G})-0.5\mathrm{log}(K)\left|{\Theta }_{\mathcal{G}}\right|\mathrm{,}$$

where ${\ell}(\mathcal{D}:\widehat{\theta },\mathcal{G})$ is the log-likelihood of the data given the estimated parameters $\widehat{\theta }$ and the graph structure $\mathcal{G}$, $\widehat{\theta }$ are the maximum-likelihood parameters, K is the number of samples and $\left|{\Theta }_{\mathcal{G}}\right|$ represents the size (e.g., the number of parameters or edges) of the parameter set $\Theta$ associated with the graph $\mathcal{G}$.

The decomposition of the likelihood in DCBN learning enables us to utilize the BIC score to learn ${\mathcal{G}}_{0}$ and ${G}_{\to }$ separately. In learning ${\mathcal{G}}_{0}$, $K$ corresponds to the number of sequences, whereas in the case of learning ${G}_{\to }$, $K$ represents the total number of transitions in all sequences.

We learn network structure using a score-based greedy hill-climbing search with local edge operations (add/delete/reverse), enforcing acyclicity and time-slice constraints (feedback is modeled across time, not within a slice). The score is BIC, which (i) is decomposable, allowing separate learning of the initial ${G}_{0}$ and transition ${G}_{\to }$ graphs, and (ii) penalizes complexity, promoting sparse, interpretable structures. Greedy search is computationally feasible for our graph size and fits the copula-based likelihood factorization used here. To mitigate local optima, we ran multiple random restarts and retained the best-scoring DAG. In sensitivity checks, replacing BIC with AIC yielded the same key arcs, indicating robustness of the learned dependencies.

We estimate copula parameters via (a) score-based local optimization with $\tau$-based initialization and 100 random restarts, and (b) Bayesian MCMC (posterior medians, 95% CrI). We enforce DAG and time-slice constraints. Convergence is assessed by ($\hat R$< 1.01) and effective sample size, with posterior predictive checks and PSIS-LOO for comparative fit. For edges showing larger dispersion, we re-screen copula families and report MCMC as primary; local estimates are retained as diagnostic values only.

We use score-based greedy hill-climbing with local add/delete/reverse moves, BIC scoring, and time-slice constraints (no within-slice cycles; cross-time edges only from $t-1$ to). Multiple random restarts and $\tau$ based initialization reduce local-optimum risk. BIC's decomposability lets us learn ${G}_{0}$ and ${G}_{\to }$ efficiently; sensitivity to AIC yielded the same key arcs.

#### Copula learning parameters

To learn the copula parameters in a DCBN, we aim to maximize the log-likelihood of the training dataset $D$ given the model $DC$.

$${\ell}(D:DC)=\sum_{m=1}^{M} \mathrm{log}\left(\prod_{t=0}^{{T}_{m}} {f}_{{\mathcal{X}}_{m}^{(t)}}\left({\mathbf{x}}_{m}^{(t)}\mid {\mathbf{x}}_{m}^{(t-1)}\right)\right)$$

This log-likelihood, denoted as ${\ell}(D:DC)$, is computed by summing all the sequences in the dataset and all the time steps within each sequence. For each time step $t$ in each sequence $m$, the log-likelihood function involves the joint density function ${f}_{{\mathcal{X}}_{m}^{(t)}}$, which represents the conditional probability density of the random variables at time $t$ given the variables at time $t-1$. The maximization of the log-likelihood is typically achieved through a combination of local optimization algorithms, such as the Newton–Raphson method, and MCMC optimization techniques, such as gradient descent or the metropolis ratio (MR) [50–52].

Marginals checked by PIT/DT histograms and slice-wise goodness-of-fit. Copulas: families are continuous/differentiable with identifiable parameters; we screen families (including tail-asymmetric types) by log-likelihood, AIC/BIC, and PSIS-LOO. Dynamics: non-stationarity is accommodated via slice-specific${\theta }_{t}$; we summarize persistence by the trend of $\tau /$ parameters across slices. Estimation: MCMC is primary, with $\hat R<1.01$, effective sample sizes, and posterior predictive checks (PPC- $\tau$) reported in Figure S.2. Identifiability is supported by the $\tau$-parameter links and bounded priors; edges with weak identifiability are flagged and interpreted cautiously. Full transformations and PPC details are provided in Supplement S10; convergence and family-sensitivity results are in Table S.10.

### Metrics of goodness of fit

In this study, we used several goodness-of-fit measures to evaluate the performance of different copula models, including the likelihood value, Akaike information criterion (AIC), Bayesian information criterion (BIC), root mean square error (RMSE), and Nash–Sutcliffe efficiency (NSE).

The likelihood value represents the probability of observing the given data $D$ under the estimated parameters $\widehat{\theta }$ and copula model $C$. In the context of DCBNs, the likelihood value indicates how well the copula model fits the observed data sequence across multiple time points. Higher likelihood values indicate better model fit.

$$\mathrm{Likelihood}(D:\widehat{\theta },C)$$

AIC balances the goodness of fit of a model with its complexity, penalizing overfitting [53].

$$\mathrm{AIC}=-2\cdot \mathrm{Likelihood}(D:\widehat{\theta },C)+2\cdot k$$

where $k$ is the number of parameters *k* in the copula model.

The BIC is similar to the AIC but incorporates a stronger penalty for model complexity [49].

$$\mathrm{BIC}=-2\cdot \mathrm{Likelihood}\left(D:\widehat{\theta },C\right)+k . \mathrm{log}(n)$$

where $n$ is the logarithm of the number of data points.

The RMSE quantifies the average difference between the observed ${y}_{i}$ and predicted ${\widehat{y}}_{i}$ values. It provides a measure of the accuracy of the copula model's predictions in DCBNs.

$$\mathrm{RMSE}=\sqrt{\frac{1}{n}\sum_{i=1}^{n} {\left({y}_{i}-{\widehat{y}}_{i}\right)}^{2}}$$

The NSE compares the observed data ${y}_{i}$ with model predictions ${\widehat{y}}_{i}$, considering the variability of the observed data. It assesses how well the copula model captures the variability and dynamics of the underlying system.

$$\mathrm{NSE}=1-\frac{\sum_{i=1}^{n} {\left({y}_{i}-{\widehat{y}}_{i}\right)}^{2}}{\sum_{i=1}^{n} {\left({y}_{i}-\overline{y }\right)}^{2}}$$

A perfect model fit is achieved with an RMSE of 0 and an NSE of 1. A higher RMSE indicates a worsening fit, while a lower NSE indicates better agreement. NSE values approaching negative infinity indicate worse predictions than the mean, while those near 1 indicate excellent agreement. Predictive fit is summarized with NSE (closer to 1 indicates better skill; 0 equals the mean predictor) and RMSE. Because RMSE is scale-dependent, we also report normalized RMSE (NRMSE = RMSE/SD_Y) and MAE/SD for comparability across edges. Dependence strength is evaluated separately using Kendall’s τ and copula parameters; these summarize association rather than point-prediction error. For this research, we utilized MATLAB R2023a, which is a powerful computational tool for data analysis and simulation purposes.

## Results

### Temporal dependencies across program participation and outcomes

As shown in Table 2, the Kendall tau for program participation (PS) → food security (FS) suggests a weak positive association at the first time slice (2009), with a tau of 0.392. This association increases in later slices (2013 and 2016) to 0.517 and 0.624, respectively, indicating moderate positive associations. This means that there is a tendency for households participating in more intensive programs (higher ranks) to also have higher ranks in food security over time. The analysis reveals a progressively stronger positive association between program participation (PS) and wealth quantile (WQ) across the three time slices. However, statistically significant Kendall rank correlations (0.588, 0.745, 0.855) suggest a trend where households participating in programs tend to have higher wealth levels over time.

**Table 2:** Kendall rank correlation coefficient (${\boldsymbol{\tau}}$) of temporal edges in the network structure

Edge	2009	2013	2016
PS → FS	0.392***	0.517***	0.624***
PS → WQ	0.588***	0.745***	0.855***
PS → MSW	0.454***	0.444***	0.828***
WQ → FS	0.561***	0.712***	0.572***
FS → CUS	−0.33***	−0.418***	−0.60***
WQ → CUS	−0.376***	−0.788***	−0.675***
MSW → CUS	0.622	0.634	0.613

^*^, **, and *** indicate significance at the 10%, 5%, and 1% levels, respectively

*FS* is oriented so that higher values indicate better food security; negative τ therefore denotes lower undernutrition with higher* FS*

Similarly, the correlation between program participation (PS) and mother subjective wellbeing (MSW) strengthened. Although statistically significant correlations (0.454, 0.444, 0.828) start weak, they become very strong by the third time slice. This increasing association coincides with the introduction of the HEP in the second time slice. The analysis reveals a positive association between the wealth quantile (WQ) and food security (FS) across the three time slices. This means that households with higher wealth (higher rank) tend to also have better food security (higher rank). While statistically significant (*p* value < 0.0001 for all), the correlations (0.561, 0.712, 0.572) suggest a moderate positive association, with some variation in strength across the time periods. The strongest link is observed in the second time slice.

Furthermore, the study revealed that the correlation between food security (FS) and child undernutrition status (CUS) increased over time. Furthermore, the study revealed that the correlation between food security (FS) and child undernutrition status (CUS) became more negative over time (τ = − 0.355, − 0.418, − 0.600), indicating that better food security is associated with lower undernutrition. In simpler terms, as food security improves, there is a decrease in child undernutrition. The association between wealth and undernutrition is negative and grows in magnitude across waves (see Table 2), consistent with lower undernutrition in wealthier households. In general, across waves, FS → CUS and WQ → CUS are negative and strengthen in magnitude, indicating that better food security and higher wealth are associated with lower undernutrition. Finally, the correlation between the mother’s subjective wellbeing and child undernutrition was not statistically significant over time.

### Persistence within outcomes (self-loops)

Over time, child undernutrition status exhibited an increasing positive association (correlation coefficients: 0.481 to 0.614). Similarly, wealthier households consistently maintained their socioeconomic status (correlation coefficients: 0.541 to 0.755). Food security status persisted from 2013 to 2016 (correlation coefficients: 0.637 to 0.846). However, the correlation of food security status between 2009 and 2013 did not reach statistical significance (see Table 3).

**Table 3:** Kendall rank correlation coefficient (${\boldsymbol{\tau}}$) of self-loop edges in the network structure

Edge	2009 → 2013	2013 → 2016
CUS → CUS	0.481***	0.614***
FS → FS	0.064	0.846***
WQ → WQ	0.541***	0.755***

^*^, **, and *** indicate significance at the 10%, 5%, and 1% levels, respectively

**Fig. 3:**
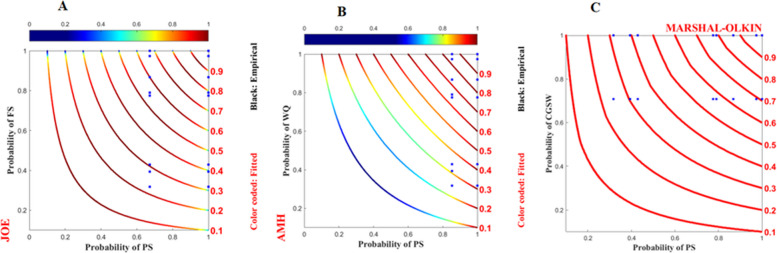
Dependence structure of programme participation on the x-axis with food security, wealth quantiles, and mother wellbeing on the y-axis are presented in probability space. The red lines represent the copula isolines, and the blue dots represent the observed data

Figure 3 shows the dependency structure between program participation and food security, wealth quantiles, and mother wellbeing. The study revealed a positive relationship between household program participation and food security. As households enroll in the initiative program, their probability of having secured food status seems to decrease initially (as indicated by the blue isoline, which was derived from the Joe copula at the top tail, indicating that a low probability of program participation leads to a low probability of food security status). However, as their participation probability increases, there is a corresponding increase in the probability of achieving food security (as evidenced by the shift from blue to red in the Ali-Mikhail-Haq (AMH) copula isoline, indicating that a high probability of program participation leads to a high probability of food security). On the other hand, as households continue participating in the program, both their food security (as depicted in Fig. 3A) and wealth status (as shown in Fig. 3B) improve. Finally, household participation in the program is crucial for improving mother' subjective wellbeing within the high probability space (as illustrated in Fig. 3C).

**Fig. 4:**
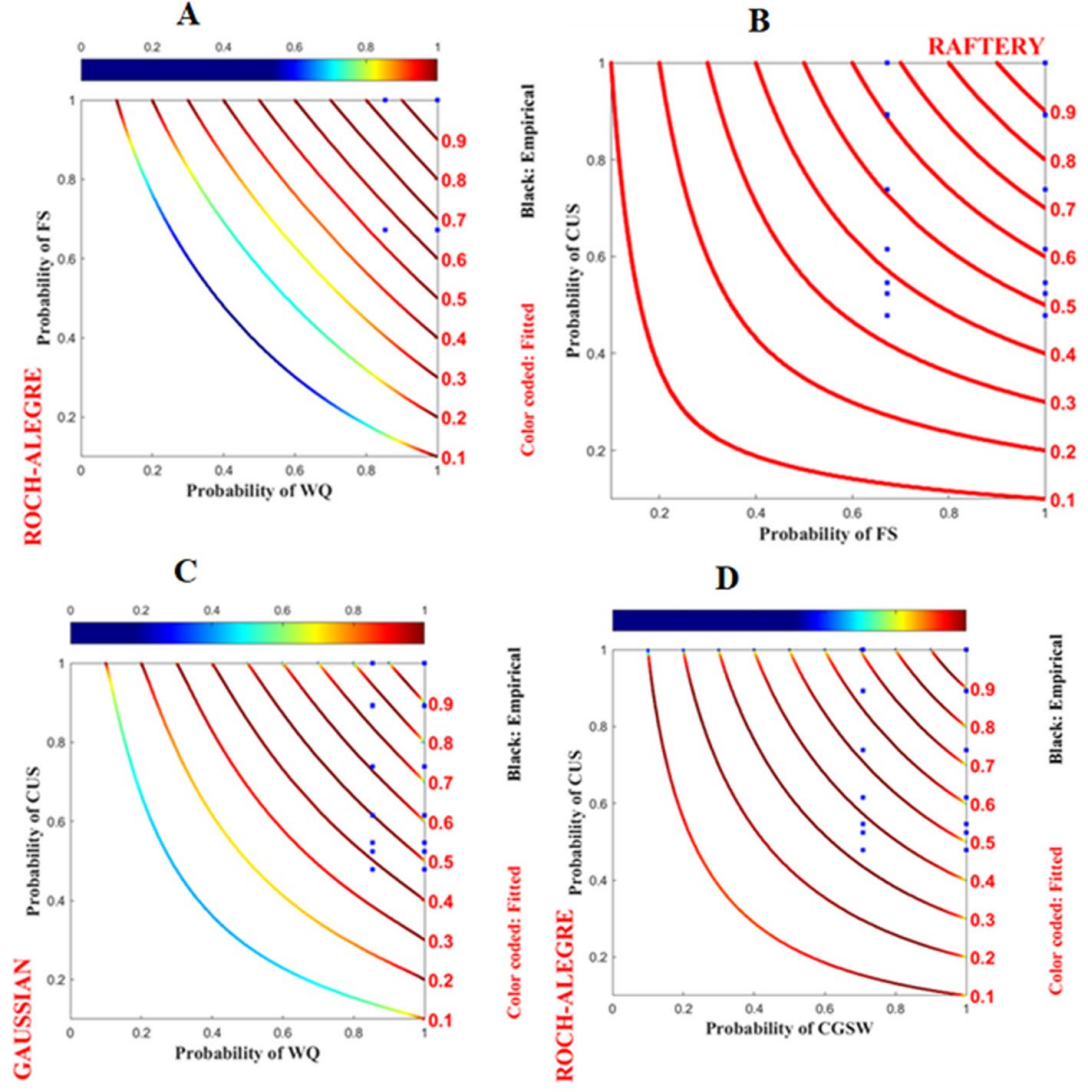
Dependence structure of food security, wealth quantile, and mother's wellbeing on the x-axis with children’s undernutrition status on the y-axis are presented in probability space. The red lines represent the copula isolines, and the blue dots represent the observed data

Similarly, as shown in Fig. 4A), as the probability of the wealth status of a household increase, the probability of its food security level tends to increase. However, the dependency between these probabilities appears to be slightly weak, as indicated by the blue probability isoline Roch–Alegre copula function (Fig. 4A). The dependency between the food security status of households and the nutritional status of their children is very strong, with a near 1 probability. The probability space derived from the Raftery copula functions is asymmetric and skewed to the right (Fig. 4B). Likewise, the dependency between household wealth status and the nutritional status of their child was somewhat weaker, as evidenced by the light blue copula isoline indicating that at low household wealth status, there is a low child nutritional status (Fig. 4C). However, the probability of dependency tends to increase as a household's wealth status increases. Finally, the dependency between mother’s subjective wellbeing and the nutritional status of their child was strong, as indicated by the red isolines of the Roch-Alegre copula function (Fig. 4D).

**Fig. 5:**
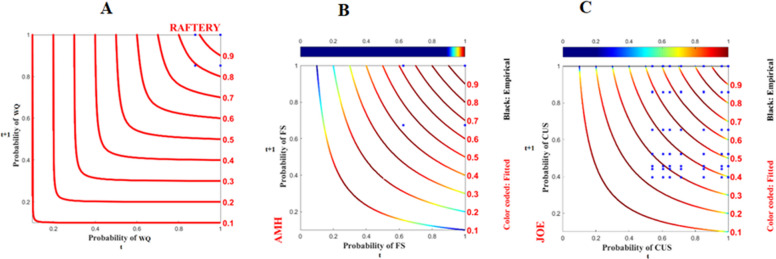
Self-dependence structure of food wealth quantiles, security, and children’s nutritional status across time are presented in probability space. The red lines represent the copula isolines, and the blue dots represent the observed data

The study reveals a strong connection between wealth across time points. Households that were wealthy at one point (t) were very likely to remain wealthy at the following point (t + 1), as shown by the red copula isoline in Fig. 5A. Likewise, households with secure food access at one point (t) were highly likely to maintain that security in the following period (t + 1) (Fig. 5B). Finally, the analysis in Fig. 5C suggests a strong dependency between a child's nutritional status at different time points. Children who were undernourished at one time point were very likely to remain undernourished at another time point.

### Copula parameter estimates and model skill

We estimate copulas from a library of 26 families, selecting the best by log-likelihood, AIC, and BIC. Table 4 reports the top-ranked family per edge and the MCMC posterior medians (95% CrI) as primary, alongside diagnostic local estimates. Model skill is summarized with RMSE (lower is better) and NSE (closer to 1 is better). For MSW → CUS, lower skill (higher RMSE, lower NSE) indicates limited predictive accuracy; we therefore interpret dependence primarily via Kendall’s τ and copula parameters rather than RMSE/NSE alone. Differences between local and MCMC for PS → MSW, WQ → FS, and WQ → CUS reflect local-optimum sensitivity; after τ-based initialization and multi-restart search, local estimates fall within the MCMC 95% CrI, and inference is unchanged. Consistent with measure orientation (higher FS/WQ = better; higher CUS = worse), FS → CUS and WQ → CUS are negative, while PS → FS, PS → WQ, and WQ ↔ FS are positive. For WQ–FS, NSE = 0.7248 indicates good predictive skill; RMSE = 0.4221 is consistent with this once expressed on a scale-free basis (NRMSE, MAE/SD, Table 4). Kendall’s τ and the selected copula parameters confirm a significant positive dependence between wealth and food security.

**Table 4:** Copula parameter estimates for key edges. Primary values are MCMC posterior medians (95% CrI). Local optimization results (τ-initialized, multi-restart) are shown for diagnostic comparison. Where local and MCMC differ, inference follows the Bayesian estimates, which passed convergence and posterior-predictive checks. Note: variable orientation is higher FS/WQ = better, higher CUS = more undernutrition; thus FS → CUS and WQ → CUS are expected to be negative

Edge	Best Copula	RMSE	NSE	NRMSE	MAE/SD	${\theta }_{1}$-Local	${\theta }_{2}$-Local	${\theta }_{1}$-MCMC	θ1-MCMC (95% CrI)		${\theta }_{2}$-MCMC	θ2-MCMC (95% CrI)	
PS → FS	Joe	0.1720	0.8263	0.4168	0.3325	1.1438***		1.1438***	1.1421	1.1453			
PS → WQ	AMH	0. 0824	0.8691	0.3618	0.2887	0.9566***		0.9566***	0.9479	0.9649			
PS → MSW	Marshal-Olkin	0.1935	0.8722	0.3575	0.2852	1.1098***	0. 2500***	0.0929***	0.0642	0.0999	0.2500***	0.2465	2.2311
WQ → FS	Roch-Alegre	0.4221	0.7248	0.5246	0.4186	0.5327***	1.1829***	0.6132***	0.5817	0.6327	1.0706***	1.0655	1.0791
FS → CUS	Raftery	0.0708	0.8264	0.4167	0.3324	0.5163***		0.5163***	0.3724	0.6909			
WQ → CUS	Gaussian	0.0754	0.8138	0.4315	0.3443	0.1406***		0.3092***	0.3096	0.3088			
MSW → CUS	Roch-Alegre	0.0083	1.000	0.0000	0.0000	0. 0010	0. 0076	0.0010	0.0009	0.1356	1.0076	1.0019	1.0078
FS → FS	AMH	0.1393	0.7931	0.4549	0.3629	0.1225***		0.1225***	0.1221	0.1230			
WQ → WQ	Raftery	0.1291	0.8453	0.3933	0.3138	0.2281***		0.2281***	0.2231	0.2331			
CUS → CUS	Joe	0.1785	0.8291	0.4134	0.3298	1.1474***		1.1474***	1.1435	1.1512			

^*^, **, and *** indicate significance at the 10%, 5%, and 1% levels, respectively. The empty cells in the table indicate that specific copulas do not have a second parameter (refer to Table S.10 in the Supplementary for further details). NSE: 1 = perfect; 0 = mean model. NRMSE = RMSE/SD(Y); using the SD of the child node Y for each edge (e.g., FS for WQ → FS). τ and copula parameters summarize dependence, not predictive error. Orientation: higher FS/WQ = better; higher CUS = worse

Discrepancies between local and MCMC estimates on PS → MSW, WQ → FS, and WQ → CUS were traced to local optima and family sensitivity. Using τ-based initialization and multiple restarts reduced gaps; local optima generally lie within the MCMC 95% CrI. Mapping parameters to τ-equivalents confirms consistent direction and magnitude across estimators (PS → FS, PS → WQ, and WQ ↔ FS positive; FS → CUS and WQ → CUS negative given our measure orientation). We therefore privilege MCMC for inference and present local estimates for transparency.

Similarly, the study revealed statistically significant positive relationships between children’s undernutrition status, food security, and household wealth status across successive time points. This suggests potential patterns of continuity in the circumstances of variables. Furthermore, the difference between the MSW and CUS did not reach statistical significance at the 5% level. This suggests that variations in the MSW itself might not have had a strong direct association on child undernutrition status in this dataset.

For PS → MSW, WQ → FS, and WQ → CUS, initial gaps between local and MCMC estimates were traced to local optima and family sensitivity. Using τ-based starts and random restarts narrowed differences; local optima generally fall within the MCMC 95% CrI. Mapping parameters to τ-equivalents shows direction and qualitative magnitude are preserved across estimators. We therefore privilege MCMC in interpretation; local values are shown for transparency (Table S.10).

### Posterior distributions and model diagnostics

**Fig. 6:**
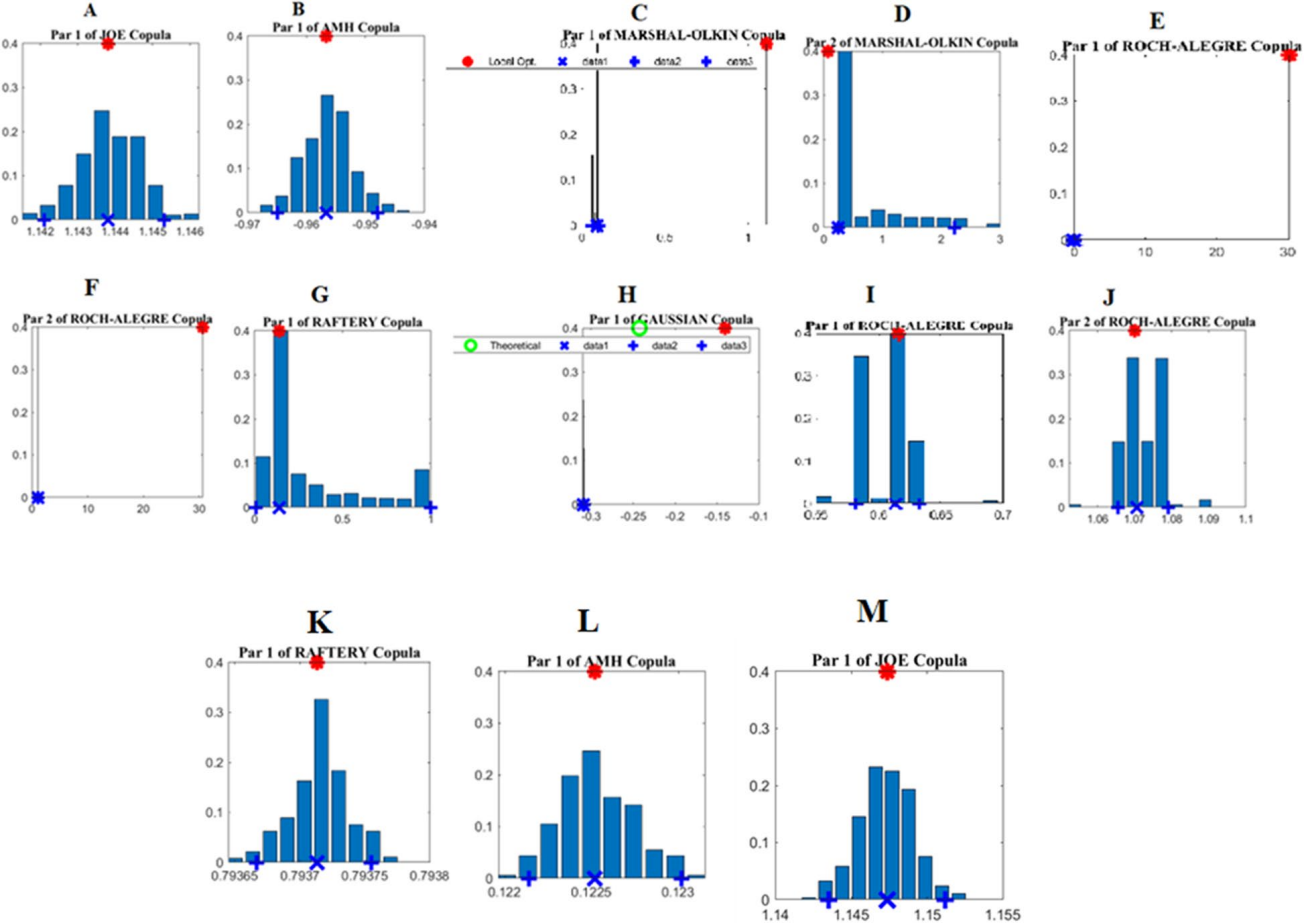
Posterior distributions of a) ${\theta }_{1}$ of Joe for PS → FS, b) ${\theta }_{1}$ of AMH for PS → WQ, c) P ${\theta }_{1}$ of Marshal-Olkin for PS → MSW, d) ${\theta }_{2}$ of Marshal-Olkin for PS → MSW, e) ${\theta }_{1}$ of Roch-Alegre for WQ → FS, f) ${\theta }_{2}$ of Roch-Alegre for WQ → FS, g) ${\theta }_{1}$ of Raftery for FS → CUS, h) ${\theta }_{1}$ of Gaussian for WQ → CUS, i) ${\theta }_{1}$ of Roch-Alegre for MSW → CUS, J) ${\theta }_{2}$ of Roch-Alegre for MSW → CUS, k) ${\theta }_{1}$ of Raftery for WQ → WQ, l) ${\theta }_{1}$ of AMH for FS → FS, and m) ${\theta }_{1}$ of Joe for CUS → CUS copulas derived from MCMC simulation within a Bayesian network. The red asterisks on top of each plot show the copula parameter values derived by the local optimization approach, whereas the blue bins are the MCMC-derived parameters, and the blue crosses show the maximum likelihood parameters of the MCMC

In this section, we refocus on the uncertainties associated with copula modeling and illustrate the posterior parameter distributions of a selection of 13 representative copula families (see Fig. 6). The posterior parameters ${\theta }_{1}$ of the Joe copula for PS → FS, ${\theta }_{1}$ of the AMH copula for PS → WQ, ${\theta }_{2}$ of the Marshal-Olkin copula for PS → MSW, ${\theta }_{1}$ of the Raftery copula for FS → CUS, ${\theta }_{1}$ of the Roch-Alegre copula for MSW → CUS, ${\theta }_{2}$ of the Roch-Alegre copulas for MSW → CUS, ${\theta }_{1}$ of the Raftery copula for WQ → WQ, ${\theta }_{1}$ of the AMH copula for FS → FS, and ${\theta }_{1}$ of the Joe copula for CUS → CUS (Fig. 6 A, B, D, G, I, J, K, L, and M) are well constrained. Notably, the copula parameters obtained through the local optimization algorithm (indicated by red asterisks atop each plot) align with the mode of the distribution (the most likely parameter, denoted by a blue cross at the bottom of each plot) derived from the MCMC simulation. However, this alignment is not consistent across all copula families, such as ${\theta }_{1}$ for the Marshal–Olkin copula for PS → MSW, ${\theta }_{1}$ for the Roch–Alegre copula for WQ → FS, ${\theta }_{2}$ for the Roch–Alegre copula for WQ → FS, and ${\theta }_{1}$ for the Gaussian copula for WQ → CUS (Fig. 6 C, E, F, and H). Therefore, these estimated parameter copula families from the local optimization algorithm diverge significantly from their counterparts obtained through the MCMC simulation.

### Sensitivity and spillover robustness

Baseline balance checks showed only modest differences between eventual participants and non-participants (maximum standardized mean difference ≈ 0.18 for WQ in 2006; Supplementary Tables S.2–S.3). Inverse probability weighting (IPW) based on pre-2009 covariates slightly attenuated Kendall’s τ values (Δτ ≈ ± 0.02) but preserved direction, magnitude, and statistical significance (Supplementary Table S.4). Similarly, copula parameter estimates under weighting remained stable, with only minor reductions in effect size (Supplementary Table S.5). These findings indicate that pre-program selection does not substantially alter the substantive conclusions.

To account for potential spillover effects, we incorporated a community program intensity (CPI) proxy as an exogenous node in the probabilistic graphical model. CPI showed consistent positive associations with food security and household wealth, and protective (negative) associations with child undernutrition. Robustness analyses using alternative CPI specifications—baseline intensity, terciles, program-specific intensity, exclusion of small clusters, and lagged CPI—yielded the same sign and direction across all edges (Supplementary Table S.7).

Finally, convergence diagnostics confirmed stable MCMC estimation across all edges (R̂ ≈ 1.00, effective sample sizes > 3,000; Supplementary Table S.9), and posterior predictive checks indicated adequate coverage of observed Kendall’s τ and marginal distributions (Supplementary Figures S2 and S3). Together, these results demonstrate that the main findings are robust to baseline selection, model specification, and community-level spillovers.

### Predictive skill with uncertainty and sensitivity

Model fit diagnostics indicate that the copula-based DCBN achieved satisfactory predictive performance across most key edges. Metrics such as RMSE, NSE, NRMSE, and MAE/SD (Table 4) confirm that dependencies like PS → FS, PS → WQ, and WQ → FS are captured with good predictive accuracy, while weaker performance is observed for MSW → CUS. In cases of modest predictive skill, inference is nevertheless supported by Kendall’s τ and copula parameter estimates, which provide robust direction and magnitude of association.

Uncertainty was explicitly assessed through Bayesian MCMC estimation. Posterior predictive checks (PPC-τ, PIT/rank histograms) demonstrate that the fitted models reproduce observed rank dependence and marginal behavior, with observed τ falling within the posterior predictive intervals for all key edges (Figures C1-C2). Convergence diagnostics (Table S.9) indicate stable chains, high effective sample sizes, and no divergences. Local estimates generally fall within MCMC 95% credible intervals (Table S.10; Figure C3), with τ-equivalents differing by ≤ 0.02, reinforcing the reliability of inference.

Together, these findings suggest that the DCBN provides a coherent and well-calibrated representation of time-varying dependencies. Even where predictive fit is modest, the direction and significance of associations remain robust, and uncertainty quantification ensures transparency in interpreting the results.

## Discussions

The study revealed that participation in combined initiative programs positively influenced household food security, wealth, and mother’s subjective well-being over time. Similar to the findings of other scholars [54, 55], the PSNP appears to provide households with consistent access to food and resources, mitigating the risk of food scarcity. Engagement in public work, as also noted in related studies, enables households to secure a steady income or food supply, which contributes to increased food availability and diversity [36, 37, 40, 56–59]. The initiative’s approach to providing aid and resources can help households save money and invest in assets. Through receiving emergency aid, families can avoid selling their assets during crises, and through health programs, they can reduce healthcare expenses, allowing for better financial management and wealth accumulation [60, 61]. Furthermore, HEP often includes nutritional education for mothers, vaccinations, and regular health check-ups for children, consequently improving the mother’s wellbeing.

Additionally, the research revealed a statistically significant positive dependency between the wealth status of households and their level of food security over time. Existing research confirms that there is a statistically significant positive relationship between a household’s wealth and food security. Households that possess greater wealth often also experience enhanced food security. This dependency can be attributed to the fact that wealthier families have the financial means to ensure a consistent and diverse supply of nutritious food, which is a fundamental component of food security. This observation aligns with research that highlights the capacity of wealthier families to invest in superior agricultural inputs and practices, which in turn bolsters food production and ensures stability [62]. Furthermore, having a higher wealth status allows households to withstand economic fluctuations and agricultural risks, such as crop failures or market volatility, thereby maintaining their food security even in challenging times [63–65].

This study revealed a significant positive dependency between household wealth and the nutritional status of children over time, suggesting that families with more economic resources tend to have children with better nutritional status. This is supported by research indicating that wealthier households can afford more nutritious diets, which are crucial for children’s growth and development [66]. Additionally, the study revealed a positive dependency between household food security and children’s nutritional status over time. Consistent access to adequate and nutritious food, which is often more available in financially stable households, is essential for the proper physical and cognitive development of children [67]. The dependency between wealth and food security is crucial; as wealth increases, it often leads to improved food security, which in turn directly benefits the nutritional health of children. These findings underscore the importance of economic stability and food availability in combating nutritional deficiencies among young people.

The study’s observation that mother wellbeing has no significant statistical dependency on the nutritional status of children over eight years old suggests that as children grow, they become more self-reliant and are influenced by a broader range of factors beyond the home environment [68, 69]. Younger children, however, are more dependent on their mother, and their nutrition is directly impacted by the mother’s wellbeing [70, 71].

The study suggested that the current state of household food security, wealth, and child nutrition has a strong influence on future status. This means that if families are doing well today in these areas, they are likely to continue on a positive trajectory. These findings show that good nutrition for children in early life not only is currently beneficial but also sets the stage for continued health and wellbeing [72–74].

Finally, our findings indicate that the MCMC optimization technique outperformed local optimization due to its efficient exploration of complex dependencies in the network and ability to converge to the true posterior distribution more effectively. This finding aligns with broader research in the field. MCMC methods are known for their ability to escape local optima and explore the global solution space more effectively, which is particularly beneficial in complex, high-dimensional problems [75]. This advantage is due to the stochastic nature of MCMC, which allows for a more thorough search of the parameter space [76]. Additionally, MCMC techniques have been shown to provide better convergence to the global optimum, especially in cases where the objective function landscape is rugged with many local optima[77]. The MCMC method also inherently quantifies uncertainty in parameter estimates, offering a robust framework for addressing the challenges of modeling dependencies in Bayesian network structures [78–80].

We explicitly incorporated pre-intervention baselines (2002/2006) to condition the initial state and to evaluate selection into programs prior to 2009. These steps mitigate, though cannot eliminate, concerns about baseline differences when formal treatment assignment is unobserved before 2009. Accordingly, we frame findings as dynamic dependencies best interpreted as robust associations within a copula-based DBN, rather than causal effects.

Consistent with theory, the negative FS → CUS dependence intensifies from 2009 to 2016, indicating that improvements in food security are increasingly associated with reductions in undernutrition.

While based on 2009–2016, our model highlights actionable levers—improving food security (FS), household wealth (WQ), and mother support—that remain central to reducing child undernutrition (CUS). The DCBN framework can be refit with contemporary datasets for routine tracking and early warning (e.g., estimating FS → CUS, WQ → CUS by region/season), enabling program managers to target areas with the highest potential impact.

### Policy implications

The findings suggest that Ethiopia’s social protection and health programs (PSNP, HEP, and EAP) have contributed to measurable improvements in food security and household wealth, which in turn reduce child undernutrition risks. Policies should therefore continue to strengthen integrated approaches that combine food transfers, income support, and community-based health services. Moreover, the DCBN framework offers policymakers a transferable monitoring tool for identifying which household factors—food security, wealth, or mother wellbeing—yield the greatest potential for reducing child undernutrition. Two actionable levers emerge: (i) strengthen FS (availability, access, diet quality) and WQ (productive assets, livelihood support), given their negative associations with CUS; and (ii) leverage community spillovers by prioritizing clusters with lower CPI or weak FS/WQ and monitoring indirect benefits.

Although our analysis is based on 2009–2016 data, the structural dependencies it reveals remain policy-salient. FS and WQ continue to be the most critical determinants of CUS in Ethiopia, and the DCBN can be refit with contemporary datasets for routine tracking and early warning. Importantly, Ethiopia’s core social protection and health initiatives (PSNP, HEP, EAP) remain in place today, even as the country faces compounding challenges from conflict, displacement, and reduced humanitarian support from donors such as UNICEF and WFP. Our findings therefore have immediate relevance: they highlight how strengthening FS, WQ, and mother wellbeing through these programs can directly reduce child undernutrition. By demonstrating the continued effectiveness of these pathways, the study provides timely evidence to sustain political commitment, encourage donor investment, and guide adaptive targeting in contexts of crisis.

### Limitation and future research

No study is without limitations. This study uses historical Young Lives waves (2009–2016) and, although we condition on 2002/2006 baselines, pre-2009 program status is unavailable; results are therefore associational, and residual selection or unmeasured community factors may remain. Spillovers are proxied with a leave-one-out community intensity under partial interference, and we lack network/geospatial data to model cross-community effects. Copula-family choice and dynamic assumptions may be mis-specified despite robustness checks, and predictive skill varies across edges. Generalizability is limited to the study sites and period. Future work should pair contemporary household data with administrative program registries to enable quasi-experimental identification (e.g., DiD/IV/synthetic control), incorporate network/geo information for richer interference, allow latent factors and spatial/multilevel DCBNs, and evaluate time-varying/vine copulas with out-of-sample validation and subgroup analyses.

## Conclusions

Participation in initiative programs is positively associated with food security (FS) and household wealth (WQ) over 2009–2016, and both FS → CUS and WQ → CUS show negative and strengthening associations—consistent with lower undernutrition as FS and WQ improve. Associations within processes (FS, WQ, CUS) persist across waves, indicating that current status is informative about near-term trajectories. These patterns remain after baseline conditioning (2002/2006), inclusion of a community spillover proxy, IPW sensitivity, and privileging MCMC over local optimization. These findings highlight the program’s impact on multiple socioeconomic factors and underscore its role in fostering holistic household development. Findings are associational and suggest practical levers for policy: prioritizing interventions that raise FS and WQ and tracking FS → CUS and WQ → CUS edges by region/season to guide targeting. Temporal dependencies underscore the importance of present household conditions in shaping children’s nutritional futures, while re-estimation with contemporary data can extend this dependence map for ongoing decision support.

## Supplementary Information

Supplementary Material 1.

## Data Availability

The dataset used in this study was obtained from the Young Lives Study. Access to the data can be obtained either by completing the form available at Young Lives Data Access, selecting the dataset "Young Lives: Rounds 1–5 constructed files, 2002–2016" (used in this study), or by creating a user account through the UK Data Service, subject to their terms and conditions. Additionally, the survey questionnaires for each round (Rounds 1–5) are available through the following link: Young Lives Round 1 Questionnaires. By adjusting the round number in the URL or navigating through the menu on the Young Lives website, users can access the questionnaires for each respective round.

## Abbreviations

AIC: Akaike information criterion
BIC: Bayesian information criterion
CPI: Community program intensity
CUS: Child undernutrition status
DAG: Directed acyclic graph
DBN: Dynamic Bayesian network
DCBN: Dynamic copula Bayesian network
EAP: Emergency aid program
FS: Food security status
HEP: Health extension program
HEWs: Health extension workers
IPW: Inverse-probability weighting
MCMC: Markov chain Monte Carlo
MSW: Mother subjective wellbeing
NSE: Nash–Sutcliffe efficiency
PS: Program participation status
PSNP: Productive safety net program
RMSE: Root mean square error
US: Undernutrition status
WQ: Wealth quantile
YL: Young lives
95% CrI: 95% Credible interval
