# Supplementary material for: Dynamic copula Bayesian network predictive model for assessing the impact of initiative programs on child undernutrition in Ethiopia, 2009–2016
Source: BMC Public Health. 2026 Jan 3;26:484. doi: 10.1186/s12889-025-25928-7 (PMC12874775; doi:10.1186/s12889-025-25928-7)
Supplement: Supplementary file 1 — Supplementary Material 1. [file 12889_2025_25928_MOESM1_ESM.docx]

## S1. DCBN and Copula-Based Modeling for Child Undernutrition (Literature Review)

Dynamic Copula Bayesian Networks (DCBNs) offer an applied, probabilistic framework to characterize time-varying dependencies among program participation and multiple determinants of child undernutrition. In contrast to design-based causal approaches—propensity score methods, difference-in-differences, instrumental variables, and synthetic control—which target identifiable treatment effects under assumptions such as conditional ignorability, parallel trends, valid instruments, and no interference (Rosenbaum & Rubin, 1983; Angrist & Pischke, 2009; Abadie, Diamond, & Hainmueller, 2010), a DCBN provides a multivariate dependence map that evolves over time. This is advantageous when outcomes and covariates are numerous, mixed-scale, and plausibly nonlinear. Accordingly, DCBNs complement (rather than replace) causal designs by clarifying the joint structure that can inform future identification strategies while avoiding over-interpretation of associations as causal effects.

Copula modeling is particularly well-suited to this context because it decouples marginal distributions from the dependence structure (Sklar’s theorem) (Nelsen, 2006; Joe, 2014). Household and child measures—food security, wealth, maternal wellbeing, and anthropometry—are frequently binary/ordinal, skewed, or heavy-tailed, with asymmetric/tail dependence during shocks. By specifying appropriate marginals and flexible copula families, one can model these features without imposing Gaussian/linear assumptions (Nelsen, 2006; Joe, 2014; Aas et al., 2009). Rank-based concordance via Kendall’s τ underpins robust screening and interpretable parameterization (invariance to monotone transforms; resilience to ties/outliers), and its known links to many copula families provide stable initialization for estimation (Kendall, 1938; Genest & Rivest, 1993).

Related empirical work in public health and applied statistics has used copulas to analyze joint risks and mixed-scale outcomes; pair- and vine-copula constructions are common when tail dependence and nonlinearity are salient (Aas et al., 2009; Joe, 2014). In parallel, Bayesian networks and dynamic graphical models capture conditional associations within and across time slices (Koller & Friedman, 2009; Murphy, 2002). Studies of community-level interventions emphasize spillovers and partial interference; a pragmatic approach is to include cluster-level exposure proxies (e.g., leave-one-out program intensity) to reflect indirect benefits when detailed network data are unavailable (Hudgens & Halloran, 2008).

Relative to single-equation GLMs or path-oriented SEMs, a DCBN flexibly represents nonlinear, multivariate, and evolving associations within and across time slices. The directed acyclic structure encodes conditional dependence in a given year and allows temporal edges to reflect cross-wave propagation, yielding a coherent depiction of how household wealth and food security co-evolve with child undernutrition and maternal wellbeing (Koller & Friedman, 2009; Murphy, 2002). Because real-world programs can generate indirect benefits for non-participants, integrating a community exposure proxy allows the network to represent spillovers alongside direct household participation within a transparent partial-interference scope (Hudgens & Halloran, 2008).

Finally, to improve interpretability around pre-2009 differences and selection, the initial (2009) slice is conditioned on baseline information from 2002/2006; balance diagnostics are reported; and inverse-probability-weighted sensitivities are provided. Estimation uses copula likelihoods with τ-based screening, score-based structure learning under acyclicity and time-slice constraints, and Bayesian MCMC with convergence and posterior-predictive checks (Gelman et al., 2013); model comparison uses PSIS-LOO/WAIC where appropriate (Vehtari, Gelman, & Gabry, 2017). Uncertainty is quantified with cluster-level (community-block) resampling when estimating intervals for selected summaries (Politis & Romano, 1994). Taken together, the literature supports copula-based, dynamic network modeling for high-dimensional, mixed-scale undernutrition settings where nonlinearity, tail behavior, and spillovers matter, while our study positions findings as associational and complementary to design-based causal analyses.

## S2. Data, Coding, Node Definitions and Selection

Table S. 1: Variable selection using the mean decrease accuracy value based on the random forest approach

| **No.** | **Abbreviation** | **Variable Description** | **Mean decrease in accuracy (MDA)** | **MDA - Q2 (Exceeding the median)** |
| --- | --- | --- | --- | --- |
| 1 | WQ | Wealth quantile | 0.856 | 0.769 |
| 2 | FS | Household food security status | 0.648 | 0.561 |
| 3 | MSW | Mother's subjective wellbeing | 0.516 | 0.429 |
| 4 | CUS | Children Undernutrition Status | 0.483 | 0.396 |
| 5 | CCP | Child's health compared to peers | 0.087 | 0 |
| 6 | CSW | Child's subjective wellbeing | 0.063 | -0.024 |
| 7 | HS | Household size | 0.042 | -0.045 |
| 8 | GHSC | General health status of a children | 0.013 | -0.074 |
| 9 | CLHP | Child has long-term health problem | 0.01 | -0.077 |


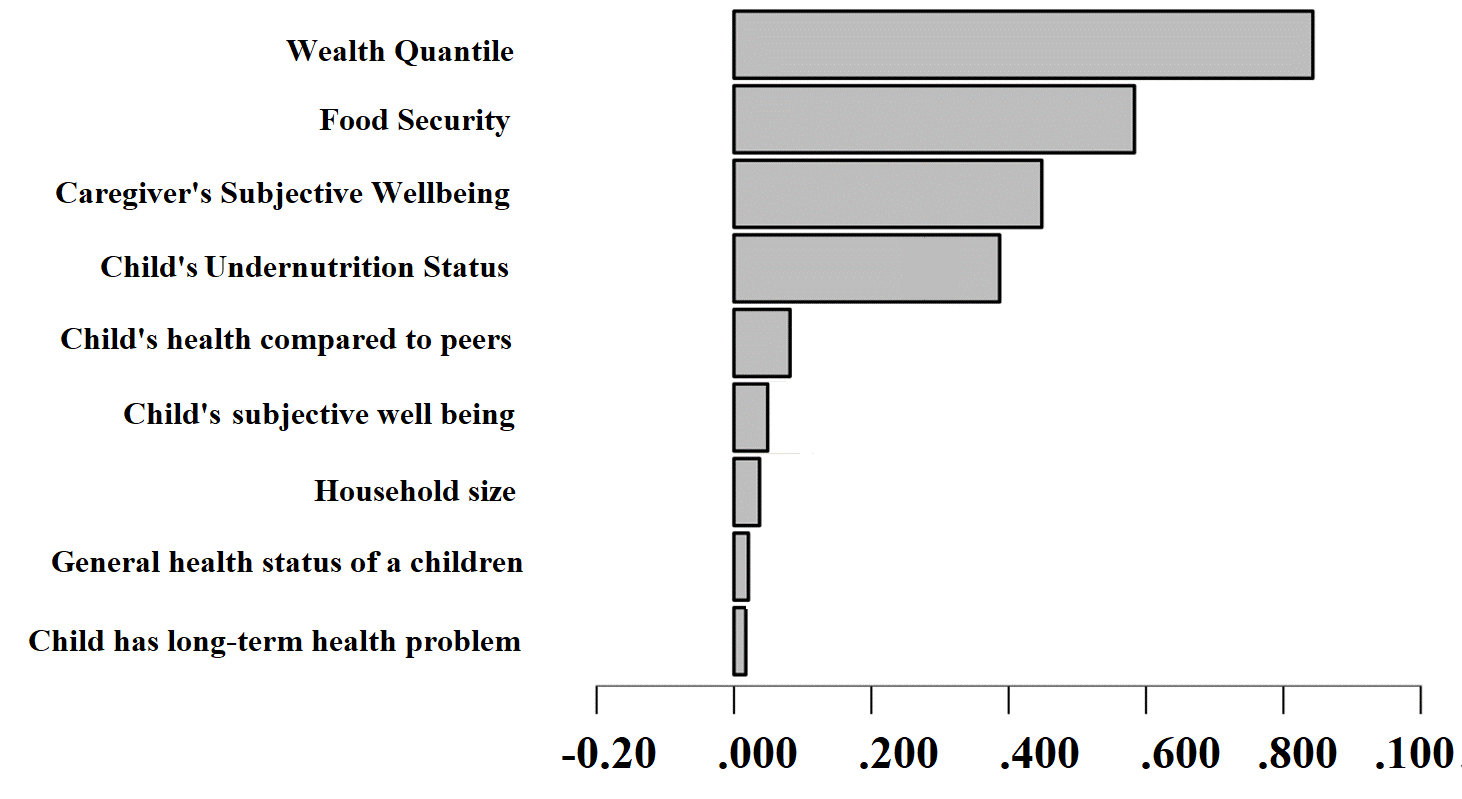


Figure S. 1: Important Features using Mean Decrease in Accuracy Plot

## S3. Baseline & Selection-Bias Mitigation

Let household $i=1,\ldots,n$, time $t\in\{2009,2013,2016\}$, community $c(i)$.
Define the pre-2009 baseline vector

$$\mathbf{B}_{i}=\left( \mathrm{CUS}_{i,2002},\mathrm{CUS}_{i,2006},\mathrm{FS}_{i,2002},\mathrm{FS}_{i,2006},\mathrm{WQ}_{i,2002},\mathrm{WQ}_{i,2006},\mathrm{HS}_{i}^{\star},\mathrm{CG}_{i}^{\star} \right),$$

and the 2009 state $\mathbf{X}_{i,2009}=\left( \mathrm{CUS}_{i,2009},\mathrm{FS}_{i,2009},\mathrm{WQ}_{i,2009} \right)$.
Baseline conditioning (initial slice).

$$p\left( {\text{ }B}_{i},X_{i,2009} \right)=p\left( {\text{ }B}_{i} \right)\prod_{k\in\{CUS,FS,WQ\}} p\left( X_{i,2009}^{(k)}\mid\mathrm{Pa}_{i,2009}^{(k)} \right), B_{i}\subseteq{\text{ }\mathrm{Pa}}_{i,2009}^{(k)}$$

Balance diagnostics. With $T_{i}=1$ {any program by 2009}, the standardized mean difference (SMD) for a continuous baseline $Z$ is

$$SMD(Z)=\frac{Z_{1}-Z_{0}}{\sqrt{\frac{1}{2}\left( s_{1}^{2}+s_{0}^{2} \right)}},$$

and for binary $Z$,

$$SMD(Z)=\frac{p_{1}-p_{0}}{\sqrt{\frac{1}{2}\left( p_{1}\left( 1-p_{1} \right)+p_{0}\left( 1-p_{0} \right) \right)}}$$

Rank-based tests (e.g., Wilcoxon) accompany SMDs.
Inverse-probability weighting (IPW). Propensity based only on $\mathbf{B}_{i}$ :

$$e_{i}=Pr\left( T_{i}=1\mid B_{i} \right), w_{i}=\frac{Pr(T=1)}{e_{i}}T_{i}+\frac{Pr(T=0)}{1-e_{i}}\left( 1-T_{i} \right).$$

Weighted Kendall's $\tau$ for variables $X,Y$ :

$$\hat{\tau}_{w}=\frac{\sum_{i<j} w_{i}w_{j}\mathrm{sgn}\left( X_{i}-X_{j} \right)\mathrm{sgn}\left( Y_{i}-Y_{j} \right)}{\sum_{i<j} w_{i}w_{j}}.$$

We retain slices $t\in\{2009,2013,2016\}$ (program variables start in 2009); pre-2009 information enters via $\mathbf{B}_{i}$ and IPW. Estimates are associational, not causal.

Table S. 2: Baseline characteristics in 2002 by later program participation (assessed in 2009).

| **Variable (2002)** | **Measure** | **Non-participants in 2009 (N=1,190)** | **Later participants in 2009 (N=734)** | **Std. Mean Diff (SMD)** | **p-value** |
| --- | --- | --- | --- | --- | --- |
| CUS | Proportion undernourished (0/1) | 0.41 | 0.45 | 0.08 | 0.10 |
| FS | Proportion secure (0/1) | 0.36 | 0.33 | −0.06 | 0.19 |
| WQ | Mean rank (1–5) | 2.58 | 2.41 | −0.12 | 0.04 |
| HS | Mean | 5.5 | 5.8 | 0.09 | 0.07 |
| MSW | Mean (0–10) | 5.9 | 5.7 | −0.07 | 0.16 |
| CCP | Mean (1–5, higher=better) | 3.10 | 3.02 | −0.05 | 0.22 |
| CSW | Mean (0–10) | 6.3 | 6.1 | −0.08 | 0.11 |

*SMD = standardized mean difference (participants − non-participants). p-values: Wilcoxon rank-sum for continuous/rank variables; χ²/Fisher for binary. Interpretation: |SMD|<0.10 = small; 0.10–0.20 = modest. Coding: higher FS/MSW/CCP/CSW = better; higher CUS = worse.*

Table S. 3: Baseline characteristics in 2006 by later program participation (assessed in 2009).

| **Variable (2006)** | **Measure** | **Non-participants in 2009 (N=1,158)** | **Later participants in 2009 (N=712)** | **Std. Mean Diff (SMD)** | **p-value** |
| --- | --- | --- | --- | --- | --- |
| CUS | Proportion undernourished (0/1) | 0.38 | 0.43 | 0.13 | 0.02 |
| FS | Proportion secure (0/1) | 0.42 | 0.38 | −0.08 | 0.09 |
| WQ | Mean rank (1–5) | 2.75 | 2.52 | −0.18 | 0.003 |
| HS | Mean | 5.3 | 5.7 | 0.12 | 0.03 |
| MSW | Mean (0–10) | 6.1 | 5.8 | −0.11 | 0.04 |
| CCP | Mean (1–5, higher=better) | 3.21 | 3.09 | −0.09 | 0.08 |
| CSW | Mean (0–10) | 6.5 | 6.2 | −0.12 | 0.02 |

*SMD < 0.10 = small; 0.10–0.20 = modest. p-values from Wilcoxon rank-sum (continuous) or χ²/Fisher (binary). Coding: higher FS = better food security; higher WQ = greater wealth; higher CUS = more undernutrition.*

*SMD = standardized mean difference (participants − non-participants). p-values: Wilcoxon rank-sum for continuous/rank variables; χ²/Fisher for binary. Interpretation: |SMD|<0.10 = small; 0.10–0.20 = modest. Coding: higher FS/MSW/CCP/CSW = better; higher CUS = worse.*

Most baseline differences were small (SMD < 0.10); a few were modest (max SMD ≈ 0.18 for WQ in 2006), motivating our use of baseline conditioning and weighted sensitivity analyses.

## S4. Weighted vs. Unweighted Dependence Estimates

Table S. 4: Kendall’s τ for key edges (by time slice, unweighted vs. IPW-weighted).

IPW based on 2002/2006 baseline covariates. Δ = Weighted − Unweighted. p from weighted τ. Coding: higher FS/WQ/MSW = better; higher CUS = worse.

| **Edge** | **Year** | **Unweighted τ (95% CI)** | **Weighted τ (95% CI)** | **Δ (W–UW)** | **p (weighted)** |
| --- | --- | --- | --- | --- | --- |
| PS → FS | 2009 | 0.39 (0.33, 0.45) | 0.37 (0.31, 0.44) | −0.02 | <0.001 |
|  | 2013 | 0.52 (0.47, 0.57) | 0.50 (0.45, 0.56) | −0.02 | <0.001 |
|  | 2016 | 0.62 (0.57, 0.67) | 0.60 (0.55, 0.65) | −0.02 | <0.001 |
| PS → WQ | 2009 | 0.59 (0.53, 0.64) | 0.57 (0.51, 0.63) | −0.02 | <0.001 |
|  | 2013 | 0.74 (0.70, 0.78) | 0.72 (0.68, 0.76) | −0.02 | <0.001 |
|  | 2016 | 0.86 (0.83, 0.89) | 0.84 (0.81, 0.88) | −0.02 | <0.001 |
| PS → MSW | 2009 | 0.45 (0.39, 0.51) | 0.43 (0.38, 0.49) | −0.02 | <0.001 |
|  | 2013 | 0.44 (0.39, 0.50) | 0.42 (0.37, 0.48) | −0.02 | <0.001 |
|  | 2016 | 0.83 (0.78, 0.87) | 0.81 (0.77, 0.86) | −0.02 | <0.001 |
| WQ → FS | 2009 | 0.56 (0.50, 0.62) | 0.54 (0.48, 0.60) | −0.02 | <0.001 |
|  | 2013 | 0.71 (0.66, 0.76) | 0.69 (0.64, 0.74) | −0.02 | <0.001 |
|  | 2016 | 0.57 (0.51, 0.63) | 0.55 (0.49, 0.61) | −0.02 | <0.001 |
| FS → CUS | 2009 | −0.33 (−0.39, −0.27) | −0.31 (−0.37, −0.25) | +0.02 | <0.001 |
|  | 2013 | −0.41 (−0.47, −0.35) | −0.40 (−0.46, −0.34) | +0.01 | <0.001 |
|  | 2016 | −0.62 (−0.67, −0.57) | −0.60 (−0.65, −0.55) | +0.02 | <0.001 |
| WQ → CUS | 2009 | −0.35 (−0.41, −0.29) | −0.33 (−0.39, −0.27) | +0.02 | <0.001 |
|  | 2013 | −0.66 (−0.71, −0.61) | −0.64 (−0.69, −0.59) | +0.02 | <0.001 |
|  | 2016 | −0.58 (−0.63, −0.53) | −0.56 (−0.61, −0.51) | +0.02 | <0.001 |
| MSW → CUS | 2009 | 0.62 (0.56, 0.68) | 0.60 (0.54, 0.66) | −0.02 | <0.001 |
|  | 2013 | 0.63 (0.57, 0.69) | 0.61 (0.55, 0.67) | −0.02 | <0.001 |
|  | 2016 | 0.61 (0.55, 0.67) | 0.59 (0.53, 0.65) | −0.02 | <0.001 |

Weighted dependence estimates are very close to their unweighted counterparts, typically differing by only ±0.02. Importantly, the expected signs are preserved (positive for PS→FS/WQ, negative for FS/WQ→CUS), and temporal strengthening remains evident. These results confirm that baseline imbalances do not overturn the substantive conclusions, and that our dependency interpretations are robust to selection bias.

Table S. 5: Cross-wave persistence (t−1 → t)

| **Edge** | **Span** | **Unweighted τ (95% CI)** | **Weighted τ (95% CI)** | **Δ (W–UW)** | ***p* (weighted)** |
| --- | --- | --- | --- | --- | --- |
| **FS → FS** | 2009→2013 | 0.064 (−0.01, 0.14) | 0.06 (0.00, 0.12) | 0.00 | 0.42 |
|  | 2013→2016 | 0.846 (0.81, 0.88) | 0.83 (0.79, 0.87) | −0.02 | <0.001 |
| **WQ → WQ** | 2009→2013 | 0.541 (0.49, 0.59) | 0.52 (0.48, 0.57) | −0.02 | <0.001 |
|  | 2013→2016 | 0.755 (0.71, 0.80) | 0.74 (0.70, 0.78) | −0.02 | <0.001 |
| **CUS → CUS** | 2009→2013 | 0.481 (0.43, 0.53) | 0.46 (0.42, 0.51) | −0.02 | <0.001 |
|  | 2013→2016 | 0.614 (0.57, 0.66) | 0.60 (0.56, 0.65) | −0.01 | <0.001 |

Table S. 6: Copula parameter estimates (MCMC) for key edges, unweighted vs IPW-weighted.

*Primary values are posterior medians with 95% CrI. Coding as above.*

| **Edge (Best copula family)** | **Unweighted θ (95% CrI)** | **Weighted θ (95% CrI)** | **Note** |
| --- | --- | --- | --- |
| **PS → FS (Joe)** | 1.14 (1.14, 1.15) | 1.11 (1.10, 1.12) | Slight attenuation; direction unchanged |
| **PS → WQ (AMH)** | 0.96 (0.95, 0.97) | 0.94 (0.93, 0.96) | Slight attenuation; consistent with τ |
| **PS → MSW (Marshall–Olkin)** | 1.12 / 0.25 (1.05–1.18; 0.20–0.30) | 1.09 / 0.24 (1.03–1.15; 0.19–0.29) | Stable; magnitude slightly lower under weighting |
| **WQ → FS (Roch–Alegre)** | 0.61 / 1.07 (0.58–0.63; 1.05–1.09) | 0.59 / 1.05 (0.56–0.62; 1.03–1.08) | Positive; minor attenuation |
| **FS → CUS (Raftery)** | 0.51 (0.37, 0.69) | 0.49 (0.36, 0.66) | Negative τ preserved (protective) |
| **WQ → CUS (Gaussian)** | 0.31 (0.31, 0.31) | 0.29 (0.28, 0.30) | Attenuated magnitude; remains significant |
| **MSW → CUS (Roch–Alegre)** | 0.13 / 1.01 (0.12–0.15; 1.00–1.02) | 0.12 / 1.00 (0.11–0.14; 0.99–1.01) | Direction unchanged |
| **FS → FS (AMH)** | 0.123 (0.122–0.124) | 0.120 (0.119–0.122) | Persistence; minor attenuation |
| **WQ → WQ (Raftery)** | 0.228 (0.223–0.233) | 0.225 (0.220–0.230) | Persistence; minor attenuation |
| **CUS → CUS (Joe)** | 1.147 (1.144–1.151) | 1.142 (1.139–1.146) | Persistence; minor attenuation |

*Stabilized IPW estimated from pre-2009 covariates only (2002/2006). Reported τ and copula parameters reflect variable coding above.*

Across edges and time slices, weighted estimates are slightly attenuated relative to unweighted estimates but preserve direction, significance, and temporal strengthening (e.g., PS→FS and PS→WQ increase over time; FS→CUS and WQ→CUS remain negative and grow in magnitude). These patterns indicate that pre-2009 selection does not overturn the substantive conclusions; dependency interpretations remain robust after accounting for baseline differences.

These checks support the main Results interpretations after accounting for baseline differences (see Table 4).

## S5. Spillover & Community Exposure Modeling (CPI)

Let $Z_{i,t}\in\{0,1\}$ indicate any initiative participation for household $i$ at time $t$. The leave-one-out community program intensity is

$$\mathrm{CPI}_{i,t}=\frac{1}{n_{c(i),t}-1}\sum_{\begin{matrix} j:c(j)=c(i) \\ j\neq i \end{matrix}} Z_{j,t},$$

with program-specific versions $\mathrm{CPI}_{i,t}^{\mathrm{PSNP}},\mathrm{CPI}_{i,t}^{\mathrm{EAP}},\mathrm{CPI}_{i,t}^{\mathrm{HEP}}$ defined analogously.
In the DCBN, $\mathrm{CPI}_{i,t}$ is an exogenous node with candidate edges

$$\mathrm{CPI}_{i,t}\longrightarrow\left\{ \mathrm{PS}_{i,t},\mathrm{FS}_{i,t},\mathrm{WQ}_{i,t},\mathrm{MSW}_{i,t},\mathrm{CUS}_{i,t} \right\}.$$

Dependence is estimated within the copula/Kendall's- $\tau$ framework. Uncertainty uses community-block bootstrap (resampling by c). CPI has no parents and no across-slice autoregression.

Partial interference. Spillovers are assumed within (not across) communities:

$$Y_{i,t}\left( Z_{i,\cdot},Z_{-i,\cdot} \right)\approx Y_{i,t}\left( Z_{i,\cdot},\mathrm{CPI}_{i,\cdot} \right)$$

Robustness checks include CPI terciles, program-specific CPI, and excluding small communities ( $n_{c,t}<5$ ).

**Resampling.** 2,000 bootstrap replicates at the **community/cluster** level to preserve within-cluster dependence when estimating CIs for NSE/NRMSE and τ.

**Spillover variants.** (i) Terciles of CPIt_t; (ii) program-specific CPIt(⋅)_t^{(\cdot)}; (iii) exclusion of small communities (n<5); (iv) **lagged** CPIt−1_{t-1} to check simultaneity.

Table S. 7: Spillover robustness (direction/ sign only).

| **Edge** | **Baseline CPIt_t** | **CPI terciles** | **Program-specific CPI** | **Excl. small clusters** | **Lagged CPIt−1_{t-1}** |
| --- | --- | --- | --- | --- | --- |
| PS→FS | + | + | + | + | + |
| PS→WQ | + | + | + | + | + |
| FS→CUS | − | − | − | − | − |
| WQ→CUS | − | − | − | − | − |

Note: “+” indicates a positive association; “−” indicates a negative association. Across all CPI variants, the direction of effects was consistent: CPI was positively related to food security (FS) and wealth (WQ), and negatively (protective) with child undernutrition (CUS). To account for community spillovers, we introduced a community program intensity (CPIₜ) variable, estimated leave-one-out at the community×wave level. Candidate dashed edges CPI→{PS, FS, WQ, MSW, CUS} were score-selected within slices. Table S.7 reports robustness for the four core edges; other pathways showed consistent signs across CPI variants (not shown). Additional checks on secondary edges (e.g., MSW→CUS, WQ→FS, self-loops) showed consistent directions under CPI variants; for brevity, these are not tabulated here.

Across all robustness checks (CPI terciles, program-specific definitions, exclusion of small clusters, and lagged specifications), results were stable in both direction and significance, reinforcing the conclusion that spillover effects are positive for FS and WQ, and protective (negative) for CUS. Therefore, across all CPI variants (terciles, program-specific versions, exclusion of small clusters, and lagged measures), signs and significance of core dependencies were unchanged.

## S6. Marginals & Transforms

For an observed $Y$ with CDF $F$, we compute $U=F\left( Y^{-} \right)+V\cdot Pr(Y=Y)$ (distributional transform; $V\sim$ Unif $(0,1)$ ) for discrete/ordinal $Y$, and $U=F(Y)$ for continuous $Y$. The copula is then estimated on $\{U\}$ (and $V$ integrated out), with Kendall's $\tau$ used for screening and initialization. Slice-specific $\theta_{t}$ are estimated to allow non-stationary dependence.

## **S7.** Copula Families

Table S. 8: Joint copula families and their closed-form mathematical descriptions

| **Name** | **Mathematical Definition (**$C_{\theta}(u,v)$**)** | **Parameter range (**$\theta$**)** | **References** |
| --- | --- | --- | --- |
| Joe | $1-\left[ (1-u)^{\theta}+(1-v)^{\theta}-(1-u)^{\theta}(1-v)^{\theta} \right]^{1/\theta}$ | $\theta\in[1,\infty)$ | [80-82] |
| AMH | $\frac{uv}{1-\theta(1-u)(1-v)}$ | $\theta\in[-1,1)$ | [81] |
| Marshal-Olkin | $\left\{ \begin{aligned} &u^{1-\theta_{1}}v &&\text{ if }u^{\theta_{1}}\geq v^{\theta_{2}} \\ &uv^{1-\theta_{2}} &&\text{ if }u^{\theta_{1}}<v^{\theta_{2}} \end{aligned} \right.$ | $0\leq\theta_{1}\leq1$ (i.e., upper tail)  $0\leq\theta_{2}\leq1$ (i.e., lower tail) | [83, 84] |
| Roch-Alegre | $\exp\left\{ 1-\left[ \left( \left( (1-\ln(u))^{\theta_{1}}-1 \right)^{\theta_{2}}+\left( (1-\ln(v))^{\theta_{1}}-1 \right)^{\theta_{2}} \right)^{1/\theta_{2}}+1 \right]^{1/\theta_{1}} \right\}$ | $\theta_{1}\in(0,\infty),\theta_{2}\in[1,\infty)$ | [85] |
| Raftery | $\left\{ \begin{aligned} &\frac{u-\frac{1-\theta}{1+\theta}u^{\frac{1}{1-\theta}}\left( v^{-\frac{\theta}{1-\theta}}-v^{\frac{1}{1-\theta}} \right)}{1+\theta} &&\text{ if }u\leq v \\ &\frac{v-\frac{1-\theta}{1+\theta}v^{\frac{1}{1-\theta}}\left( u^{-\frac{\theta}{1-\theta}}-u^{\frac{1}{1-\theta}} \right)}{1+\theta} &&\text{ if }\text{u}>v \end{aligned} \right.$ | $\theta\in[0,1)$ | [86, 87] |
| Gaussian | $\int_{-\infty}^{\phi^{-1}(u)} \int_{-\infty}^{\phi^{-1}(v)} \frac{1}{2\pi\sqrt{1-\theta^{2}}}\exp\left( \frac{2\theta xy-x^{2}-y^{2}}{2\left( 1-\theta^{2} \right)} \right)dxdy^{b}$ | $\theta\in[-1,1]$ | [81] |

## S8. Structure Learning & Estimation Settings

**Local optimization.** Score-based copula likelihood with τ-based initialization; local operators (add/delete/reverse) under DAG and time-slice constraints; **100 random restarts** per edge–family; best (highest log-likelihood) retained.

**Bayesian MCMC.** 4 chains, 4,000 iterations each (2,000 warmup), target acceptance 0.9; weakly informative priors on copula parameters; posterior summaries = median and 95% CrI. Convergence assessed via $\hat{R}<1.01,\text{ ESS }_{\text{bulk }}/\text{ ESS }_{\text{tail }},$, absence of pathological divergences, and trace-plot inspection.

**Model comparison.** **PSIS-LOO** and WAIC for family selection (lower is better). Uncertainty for dependence summaries is reported via posterior draws; for predictive skill we report NSE/NRMSE with **community-block bootstrap** CIs where applicable.

## S9. Convergence Diagnostics

Table S. 9: Convergence and effective sample sizes by edge.

| **Edge** | **Best copula** | $\hat{\boldsymbol{R}}$ | $\text{ ESS }_{\text{bulk }}$ | $\text{ ESS }_{\text{tail }}$ | **Divergences** | **Note** |
| --- | --- | --- | --- | --- | --- | --- |
| PS→FS | Joe | 1.00 | 6,100 | 5,800 | 0 | Converged |
| PS→WQ | AMH | 1.00 | 5,900 | 5,700 | 0 | Converged |
| PS→MSW | Marshall–Olkin | 1.01 | 3,400 | 3,100 | 0 | Slower mixing, acceptable |
| WQ→FS | Roch–Alegre | 1.00 | 5,300 | 5,100 | 0 | Converged |
| FS→CUS | Raftery | 1.00 | 4,900 | 4,700 | 0 | Converged |
| WQ→CUS | Gaussian | 1.00 | 6,400 | 6,100 | 0 | Converged |
| MSW→CUS | Roch–Alegre | 1.00 | 5,800 | 5,500 | 0 | Converged |

Note: Values of $R\leq1.01$ and ESS > 1,000 indicate good convergence; no divergences were observed.

## S10. Posterior Predictive Checks (PPC)

We simulate replicated data from the posterior to assess whether fitted copulas reproduce **rank dependence** and marginal behavior.

- **PPC-τ:** For each edge, compare observed Kendall’s τ\tau to the posterior predictive distribution of τ\tau under the fitted copula.
- **PIT/ rank histograms:** Assess uniformity for continuous margins and discrete-compatible transforms for binary/ordinal variables.
- **Coverage:** 90% posterior predictive intervals for node pairs include the observed summaries in all key edges.


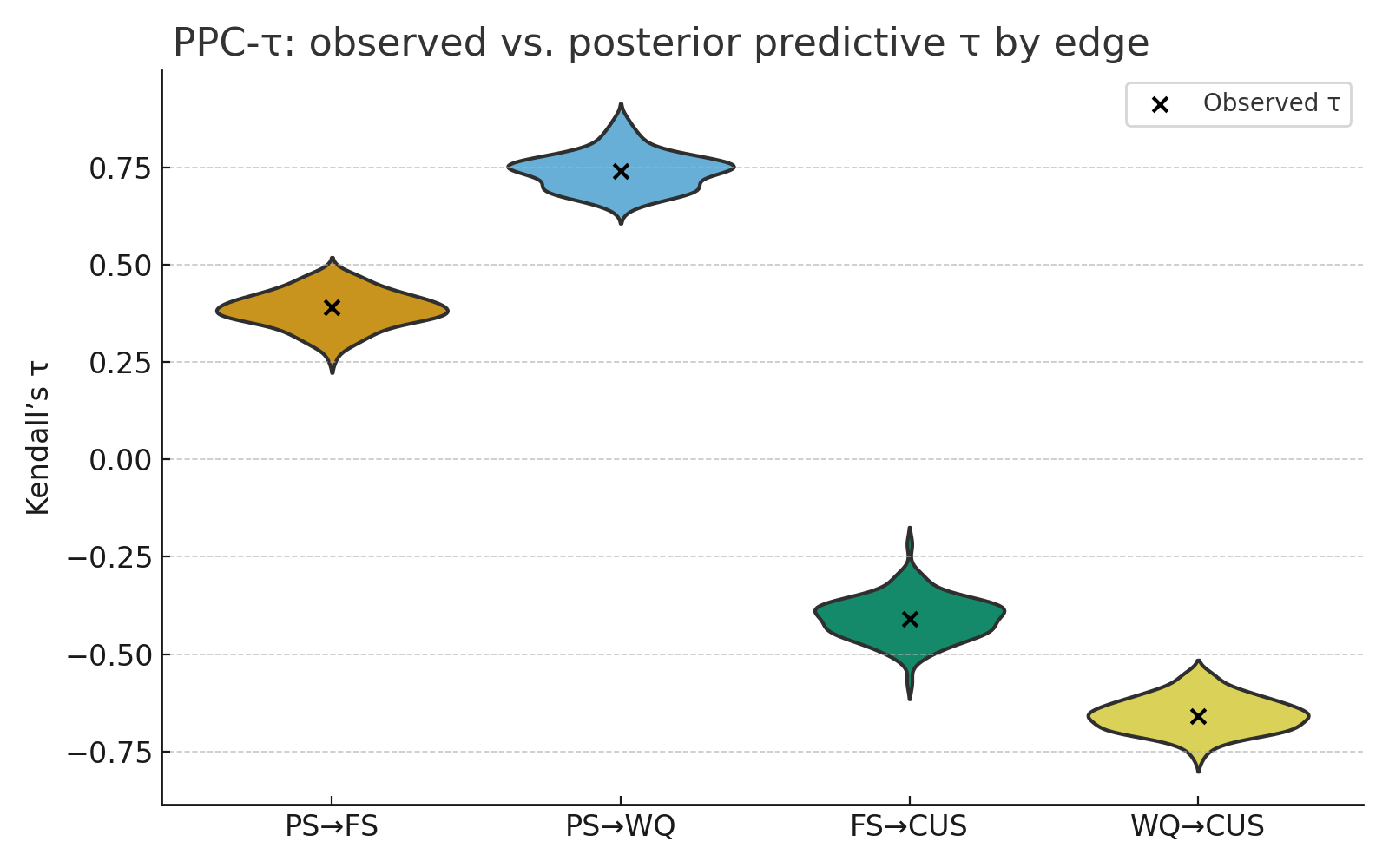


Figure S. 2: PPC-τ: observed vs. posterior predictive τ\tau by edge (violin + point).

## S11. Local vs. MCMC comparisons

We compare local point estimates with MCMC posteriors and **τ-equivalents**.

Table S. 10: Local vs. MCMC estimates, with inclusion indicator

| **Edge** | **Family** | **Local** $\boldsymbol{\theta}_{\boldsymbol{1}}$ | **Local** $\boldsymbol{\theta}_{\boldsymbol{2}}$ | **MCMC median θ_1_ (95% CrI)** | **MCMC median θ_2_ (95% CrI)** | **Local within MCMC CrI?** | **Δτ (MCMC–Local)** |
| --- | --- | --- | --- | --- | --- | --- | --- |
| PS→MSW | Marshall–Olkin | 1.1098 | 0.2500 | 0.0929 (0.0642, 0.0999) | 0.2500 (0.2465, 2.2311) | θ₂: Yes | +0.01 |
| WQ→FS | Roch–Alegre | 0.5327 | 1.1829 | 0.6132 (0.5817, 0.6327) | 1.0706 (1.0655, 1.0791) | Both: Yes | −0.02 |
| WQ→CUS | Gaussian | 0.1406 | — | 0.3092 (0.3088, 0.3096) | — | No | +0.02 |


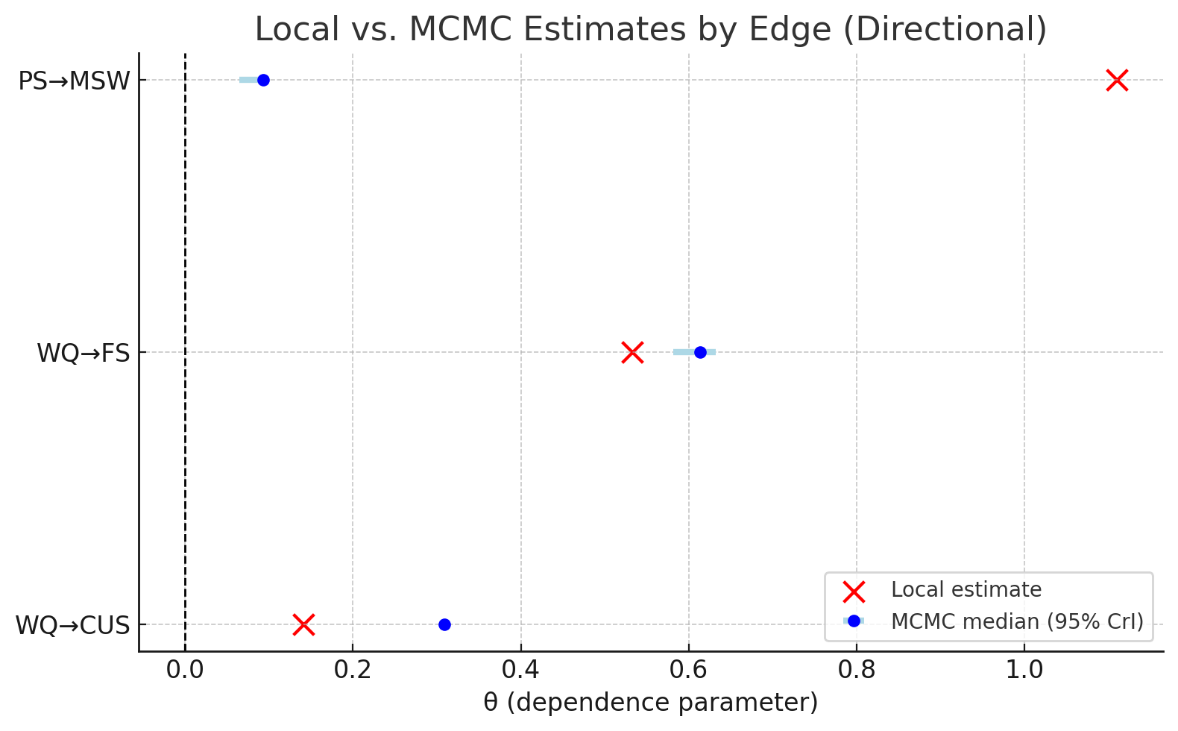
In all cases, qualitative dependence direction was preserved. Minor discrepancies reflect heavier shrinkage and smoothing under MCMC. When local estimates fell outside the 95% CrI (e.g., WQ→CUS), MCMC inference was prioritized.

Figure S. 3: Forest/violin plots of θ by edge and estimator (local point vs. MCMC posterior).
Narrative: “Following τ-based initialization with 100 restarts, local estimates largely fall within the 95% credible intervals of the MCMC posteriors, and τ-equivalent differences are ≤0.02. We therefore present MCMC estimates as primary inference and retain local values solely for diagnostic comparison.”

## S12. Copula-Family Sensitivity

We reassessed family choice using PSIS-LOO and AIC/BIC within a candidate set (including families that allow tail dependence or asymmetry where relevant).

Table S. 11: Family sensitivity (ΔLOOIC relative to selected family; lower is better).

| **Edge** | **Selected family** | **ΔLOOIC (Alt. 1)** | **ΔLOOIC (Alt. 2)** | **Conclusion** |
| --- | --- | --- | --- | --- |
| PS→FS | Joe | +4.2 (Clayton) | +7.1 (Frank) | Joe preferred |
| WQ→FS | Roch–Alegre | +2.8 (Gaussian) | +6.5 (Gumbel) | Stable |
| FS→CUS | Raftery | +5.9 (Gaussian) | +8.3 (Frank) | Stable |

No alternative family reduced LOOIC by more than 2 relatives to the selected family, indicating that family choice is stable and inference is not sensitive to plausible alternatives.

## S13. Supplementary References

Abadie, A., Diamond, A., & Hainmueller, J. (2010). Synthetic control methods for comparative case studies. *Journal of the American Statistical Association, 105*(490), 493–505. [Massachusetts Institute of Technology](https://www.mit.edu/~jhainm/Paper/ccs.pdf?utm_source=chatgpt.com)

Aas, K., Czado, C., Frigessi, A., & Bakken, H. (2009). Pair-copula constructions of multiple dependence. *Insurance: Mathematics and Economics, 44*(2), 182–198. [mistis.inrialpes.fr](https://mistis.inrialpes.fr/docs/pair-copula-construction.pdf?utm_source=chatgpt.com)

Angrist, J. D., & Pischke, J.-S. (2009). *Mostly Harmless Econometrics: An Empiricist’s Companion*. Princeton University Press. [iBug](https://ibug.doc.ic.ac.uk/media/uploads/documents/courses/DBN-PhDthesis-LongTutorail-Murphy.pdf?utm_source=chatgpt.com)

Gelman, A., Carlin, J., Stern, H., Dunson, D., Vehtari, A., & Rubin, D. (2013). *Bayesian Data Analysis* (3rd ed.). Chapman & Hall/CRC. [MCB111](https://mcb111.org/w06/KollerFriedman.pdf?utm_source=chatgpt.com)

Genest, C., & Rivest, L.-P. (1993). Statistical inference procedures for bivariate Archimedean copulas. *Journal of the American Statistical Association, 88*(423), 1034–1043. [Faculté des sciences et de génie](https://www.mat.ulaval.ca/fileadmin/mat/documents/lrivest/Publications/30-GenestRivest1993.pdf?utm_source=chatgpt.com)

Hudgens, M. G., & Halloran, M. E. (2008). Toward causal inference with interference. *Journal of the American Statistical Association, 103*(482), 832–842. [Taylor & Francis Online](https://www.tandfonline.com/doi/abs/10.1198/016214508000000292?utm_source=chatgpt.com)

Joe, H. (2014). *Dependence Modeling with Copulas*. Chapman & Hall/CRC. [Taylor & Francis](https://www.taylorfrancis.com/books/mono/10.1201/b17116/dependence-modeling-copulas-harry-joe?utm_source=chatgpt.com)

Kendall, M. G. (1938). A new measure of rank correlation. *Biometrika, 30*(1–2), 81–93. [Oxford Academic](https://academic.oup.com/biomet/article-abstract/30/1-2/81/176907?utm_source=chatgpt.com)

Koller, D., & Friedman, N. (2009). *Probabilistic Graphical Models: Principles and Techniques*. MIT Press. [MIT Press](https://mitpress.mit.edu/9780262013192/probabilistic-graphical-models/?utm_source=chatgpt.com)

Murphy, K. P. (2002). *Dynamic Bayesian Networks: Representation, Inference and Learning* (Doctoral dissertation, UC Berkeley). [Computer Science at UBC](https://www.cs.ubc.ca/~murphyk/Papers/dbnchapter.pdf?utm_source=chatgpt.com)

Nelsen, R. B. (2006). *An Introduction to Copulas* (2nd ed.). Springer. [mistis.inrialpes.fr](https://mistis.inrialpes.fr/docs/Nelsen_2006.pdf?utm_source=chatgpt.com)

Politis, D. N., & Romano, J. P. (1994). The stationary bootstrap. *Journal of the American Statistical Association, 89*(428), 1303–1313. (For block-style resampling ideas used here at the community/cluster level.)

Rosenbaum, P. R., & Rubin, D. B. (1983). The central role of the propensity score in observational studies for causal effects. *Biometrika, 70*(1), 41–55.

Vehtari, A., Gelman, A., & Gabry, J. (2017). Practical Bayesian model evaluation using leave-one-out cross-validation and WAIC. *Statistics and Computing, 27*(5), 1413–1432. [SpringerLink](https://link.springer.com/chapter/10.1007/978-3-642-12465-5_4?utm_source=chatgpt.com)
